# Efficacy and safety of Tripterygium wilfordii multiglucoside for idiopathic membranous nephropathy: a systematic review with bayesian meta-analysis

**DOI:** 10.3389/fphar.2023.1183499

**Published:** 2023-08-02

**Authors:** Hongyun Wang, Hongyan Liu, Xue Xue, Qiong Wang, Jun Yuan

**Affiliations:** ^1^ Hubei University of Chinese Medicine, Wuhan, China; ^2^ Department of Nephrology, Renmin Hospital of Wuhan University, Wuhan, China

**Keywords:** Tripterygium wilfordii Hook.f. (Celastraceae), membranous nephropathy, immunosuppression, adverse effects, network meta-analysis

## Abstract

**Background:** Currently, the optimal therapy plan for idiopathic membranous nephropathy (IMN) remains controversial as there has been no comprehensive and systematic comparison of therapy plans for IMN. Therefore, in this study, a Bayesian meta-analysis was used to systematically evaluate the clinical efficacy and safety of various intervention plans involving traditional Chinese medicine TWM in the treatment of IMN.

**Methods:** An electronic search in 7 databases was conducted from their inception to August 2022 for all published randomized controlled trials (RCTs) of various intervention plans for IMN. Network meta-analysis (NMA) was performed by using software R, and the surface under the cumulative ranking area (SUCRA) probability curve was plotted for each outcome indicator to rank the efficacy and safety of different intervention plans.

**Results:** A total of 30 RCTs were included, involving 13 interventions. The results showed that (1) in terms of total remission (TR), ① GC + CNI + TWM was the best effective among all plans, and the addition and subtraction plan of CNI + TWM was the best effective for IMN; ② All plans involving TWM were more effective than GG; ③ Among monotherapy plans for IMN, TWM was more effective distinctly than GC, while TWM and CNI were similarly effective; ④ Among multidrug therapy plans for IMN, the addition of TWM to previously established therapy plans made the original plans more effective; ⑤The efficacy of combining TWM with other plans was superior to that of TWM alone. (2) In terms of lowering 24 h-UTP, GC + TWM was the best effective and more effective than TWM. (3) In terms of safety, there was no statistically significant difference between all groups. However, CNI + TWM was the safest. No serious adverse events (AEs) occurred in all the included studies.

**Conclusion:** The addition of TWM may be beneficial to patients with IMN. It may enhance the efficacy of previously established treatment protocols without leading to additional safety risks. In particular, GC + CNI + TWM, GC + TWM, and CNI + TWM with better efficacy and higher safety can be preferred in clinical decision-making as the therapy plans for IMN.

## 1 Introduction

Idiopathic membranous nephropathy (IMN) is an autoimmune disease that mainly follows *in situ* complex formation caused by the M-type phospholipase A2 receptor (PLA2R) antigen inducing antibody (Garcia-Vives et al., 2019; Jiang et al., 2020; Liu et al., 2020). IMN is characterized by the diffuse deposition of immune complexes under the glomerular basement membrane epithelium with diffuse thickening of the basement membrane. The clinical manifestations are massive proteinuria and hypoproteinemia (Liu et al., 2022). This disease is one of the most common pathological types of nephrotic syndrome. Although proteinuria remission occurs spontaneously in about one-third of patients, IMN can still progress to end-stage renal disease (ESRD) in about 30%–40% of patients within 5–15 years, resulting in high medical costs (Rozenberg et al., 2018). In China, the prevalence of IMN has increased from 6.48% in 1999 to 22.79% in 2017 and has remained high in recent years (Jiang et al., 2020). Therefore, based on the severity of IMN, developing an appropriate treatment for IMN patients as early as possible is clinically important for controlling the progression of IMN, improving the survival quality of patients, and reducing medical costs.

The current clinical plans to treat IMN mainly are as follows: 1. Non-immunosuppressive therapies (CON): low-salt diet, anticoagulation, lipid regulation, and the application of RAAS blocking agents. 2. Glucocorticoids (GC): prednisone, methylprednisolone, etc. 3. Immunosuppressants (ISD): calcineurin inhibitors (CNI) like cyclosporine (CsA) or tacrolimus (TAC); alkylating agents (CAA) like cyclophosphamide (CTX); anti-proliferative agents like mycophenolate mofetil (MMF) and leflunomide (LEF) (Wu et al., 2021). 4. Herbal preparations: Tripterygium wilfordii multiglycosides, Huangkui capsule, etc.

GC and immunosuppressants play a role in controlling proteinuria and improving renal function in IMN patients, but these immunosuppressants commonly used in clinical practice have certain limitations in use due to their limited scope of application and toxic side effects. Hence, exploring more ideal therapy plans and giving full play to the complementary/alternative role of traditional Chinese medicine (TCM) are very necessary for improving symptoms, minimizing the toxic side effects of modern medical treatment regimens, enhancing efficacy, and reducing recurrence. Tripterygium wilfordii multiglycoside (TWM) is an active ingredient extracted from *Tripterygium wilfordii* Hook. f. [Celastraceae] (Tripterygium wilfordii Celastraceae, 2023), a plant of the Weigelaeaceae family. It has been used in the clinical treatment of immune kidney disease for more than 40 years. Its main active ingredient is triptolide (TPL), which has immunosuppressive, anti-inflammatory, and anti-tumor effects (Wang et al., 2018; Guo et al., 2021). TWM is believed to apparently improve clinical efficacy and safety in the treatment of IMN (Wang et al., 2018; Shang et al., 2018; Guo et al., 2021; Gao et al., 2021; Geng et al., 2022), but it is still not conclusive which immunosuppressive agent is more effective in combination with TWM.

Network meta-analysis (NMA) is a novel method for clinical data integration that allows simultaneous direct and indirect comparisons and ranking of superiority to assess the efficacy of multiple treatments (Lumley, 2002). This method is characterized by a large number of included studies, a large amount of data, multiple levels, complex intrinsic structure, and high interconnectedness (Lumley, 2002; Bafeta et al., 2013). Therefore, in this paper, the effectiveness and safety of various intervention plans incorporating TWM in the treatment of IMN were compared by using the systematic review and NMA, in the hope of providing a reference for clinical medication.

## 2 Materials and methods

Our study followed the PRISMA (Preferred Reporting Items for Systematic Reviews and Meta-Analyses) statement (Page et al., 2021). In addition, our study was registered on the International Prospective Register of Systematic Reviews (PROSPERO: CRD 42022355643) (Wang et al., 2022).

### 2.1 Literature search strategy

PubMed, Embase, The Cochrane Library, CNKI, WanFang, CBM, and VIP databases were searched from database inception to August 2022 for RCTs on different drug treatments for IMN, with subject terms and free-text words. In addition, the references of the included literature were also reviewed to avoid missing potentially eligible studies. Major terms used to build the search strategy included: “Glomerulonephritis, Membranous”, “Tripterygium”, etc. Specific search strategies were shown in Supplementary File S1. Only Chinese or English literature was included.

### 2.2 Literature inclusion and exclusion criteria

Inclusion criteria: (1) Study type, RCTs on different intervention plans for IMN; (2) Study subject, patients ≥18 years old who met the diagnostic criteria for IMN (diagnosis evidenced by renal biopsy or given by a physician); (3) Primary outcome measures reported based on the 2021 KDIGO guideline (Kidney Disease: Improving Global Outcomes KDIGO Glomerular Diseases Work Group, 2021): Complete remission (CR): 24-h urine total protein (24 h-UTP) quantification <0.30 g or urine protein/urinary protein creatinine ratio (uPCR) <300.00 mg/g or <30.00 mg/mmol, confirmed by two tests with an interval of at least 1 week between tests, along with normal serum albumin concentration and serum creatinine (Scr); Partial remission (PR): 24 h-UTP of 3.50g/24 h or less or uPCR <3,500.00 mg/g or <350.00 mg/mmol, 50% or more reduction from the peak value, confirmed by two tests with an interval of at least 1 week between tests, accompanied by the rebound or normalization of serum albumin concentration and stable Scr level. Total remission (TR) = CR + PR. (4) Interventions: The efficacy of monotherapy (immunosuppressant, TWM, and GC) and combination of any 2 or more of the above drugs were compared; the study must focus on at least two therapy plans, with the duration of pharmacological intervention ≥3 months. (5) Outcome measures: TR; 24 h-UTP; incidence of AEs: incidence of infection, reduced white blood cell count, elevated transaminases, etc. Exclusion criteria: (1) Non-Chinese and -English literature; (2) Some special types of literature, including reviews, case reports, basic animal experiments and theoretical discussions or empirical analyses; (3) Duplicate publications; (4) Literature whose full text is not available, whose data cannot be extracted, are incomplete or have serious errors, and that has no relevant outcome measures; (5) Literature whose study subjects have other serious diseases, such as chronic cardiovascular diseases, serious infections, and tumors; and (6) Literature on single-arm trials.

### 2.3 Literature screening and data extraction, quality evaluation

Two investigators (HYW and HYL) independently screened the literature, extracted data, and cross-checked the data according to the pre-defined inclusion and exclusion criteria. Any disagreement was resolved through discussion or consultation with a third investigator (JY). After excluding duplicates, primary screening was performed by reading the titles and abstracts of the literature to exclude apparently irrelevant literature; the screened literature was then read in full to obtain the finally included literature. For duplicate studies, studies with a larger number of study subjects and a longer follow-up period were selected. The extracted information included: basic characteristics of the included literature (first author, year and country of publication), study subject (mean age, sex ratio, sample size, basic information on subjects and baseline status such as 24 h-UTP and Scr), interventions (intervention/control measures and duration of treatment and follow-up), and key elements of risk of bias evaluation, outcome indicator data (TR, 24 h-UTP and AEs). If needed, efforts were made to contact the authors of the original study by phone or email to obtain information that was not identified but was important for this study. The quality of the final included literature was then assessed by two investigators (HYW and HYL) using the risk of bias assessment tool recommended by the official Cochrane handbook (Cumpston et al., 2019), and the results were cross-checked. Any disagreement was resolved through discussion or consultation with a third investigator (JY). The assessment included: randomization method and allocation concealment; blinding of investigators and patients; blinding of outcome measures; completeness of outcome data; selective reporting; and other risks of bias such as financial support from drug companies. The risk of bias of each item was classified as: low risk, unclear risk, and high risk, and the final results were presented in risk of bias maps by using software RevMan 5.4.1 to evaluate the quality of the included literature.

### 2.4 Statistical methods

Software R (version 4.1.3) was applied to call the gemtc package (version v1.0-1) for data analysis. Network evidence maps for NMA were first drawn, and a Bayesian model NMA was performed to compare direct and indirect evidence of the interventions included in the study. The model adopted 4 Markov chains, and the initial value was set at 2.5. 5,000 pre-iterations were performed for annealing, and 20,000 iterations were continued to reach model convergence. The model convergence was suggested to be satisfactory when the potential scale reduction factor (PSRF) tended to 1; otherwise, more iterations were needed. The probability of each intervention being the most effective treatment was determined according to the posterior probability ranking of interventions obtained under the Bayesian model. Risk ratios (RRs) and their 95% confidence intervals (CrI) were used as effect size indicators for TRs and AEs, and standardized mean differences (SMDs) and 95% CIs were used as effect size indicators for 24 h-UTP. The efficacy of each drug intervention plan was assessed by probability ranking plots, SUCRA probability curve, and the league table. Analysis of heterogeneity (ANOHE) was used to calculate the *I* ^2^ statistic to test the heterogeneity across the studies. When the overall *I* ^2^ ≤ 50%, the heterogeneity between studies was considered small or non-existent, and the fixed-effects model was used; conversely, the random-effects model was used. When there was a closed loop, nodal analysis was adopted to detect the inconsistency between direct and indirect evidence and calculate the difference and Bayesian *P*. A *p* > 0.05 indicated consistency. For the comparative studies with large heterogeneity, the sensitivity analysis of the included studies was excluded one by one, and the results were compared to evaluate the influence of heterogeneity on the stability and reliability of the synthesis results. Funnel plots for publication bias were drawn when the number of trials included in a set of comparisons exceeded 10.

## 3 Results

### 3.1 Literature search

**FIGURE 1:**
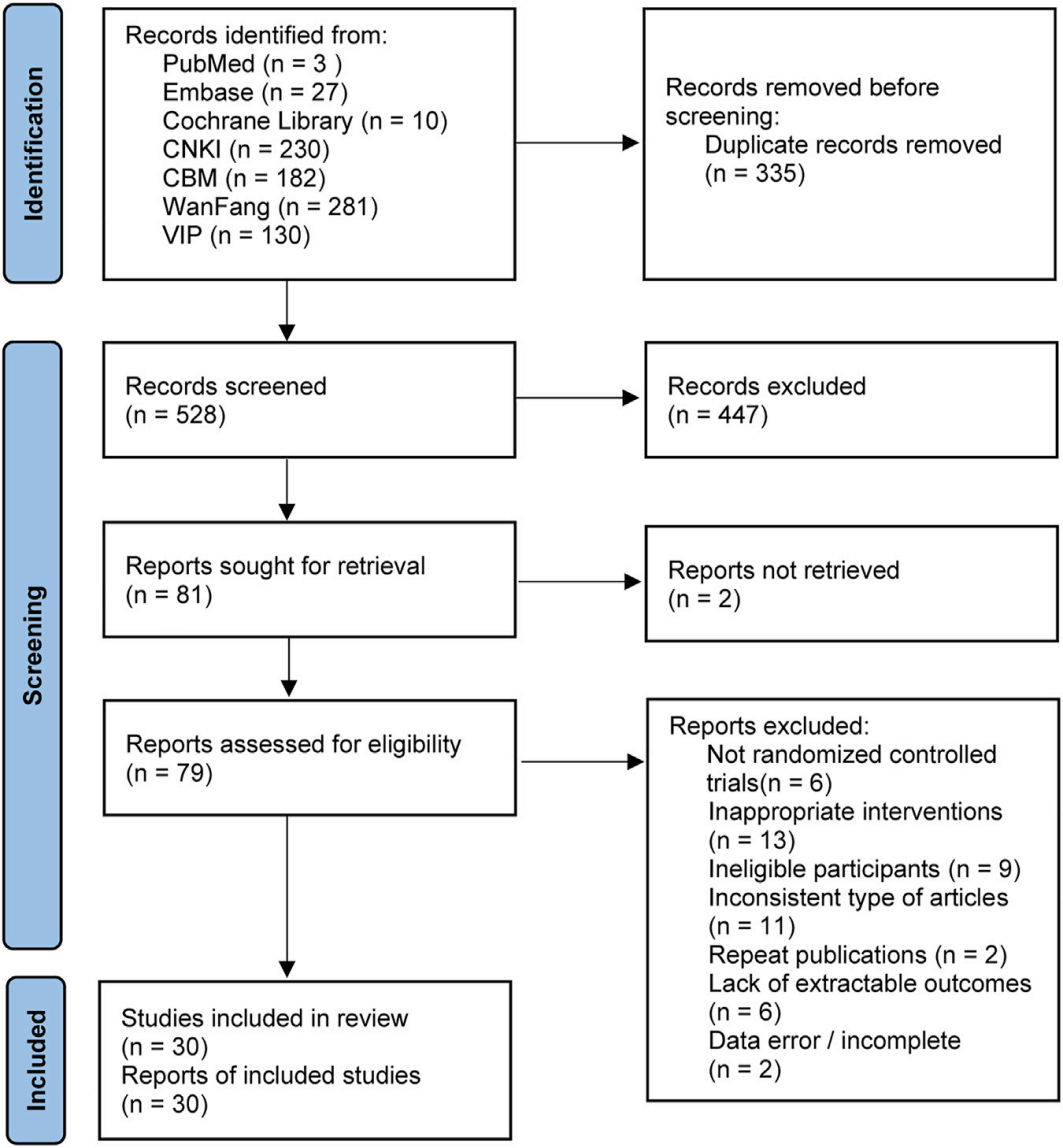
PRISMA 2020 flow diagram for new systematic reviews which included searches of databases and registers only.

The initial search yielded 863 publications, including 230 from CNKI, 281 from WanFang, 130 from VIP, 182 from CBM, 10 from Cochrane Library, 3 from PubMed, and 27 from Embase. After excluding duplicates and reading the title, abstract, and full text, 30 studies (Liu et al., 2009; Feng et al., 2011; Sun and He, 2012; Ma et al., 2014; Qiu et al., 2014; Zuo et al., 2014; Peng et al., 2015; Yang et al., 2015; Wang et al., 2016; Guo et al., 2017; Wang, 2017; Zhang, 2017; Gong, 2018; Liu et al., 2018; Yu et al., 2018; Mo et al., 2019; Xue et al., 2019; Bao, 2020; Feng, 2020; Liang et al., 2020; Wang, 2020; Xiong and Huang, 2020; Zhang et al., 2020; Zhou et al., 2020; Zhu et al., 2020; Ding, 2021; Wang, 2021; Xie and Xu, 2021; Ai and Wang, 2022; Chen et al., 2022) were finally included in the NMA, with a total of 2,410 patients. The specific literature search process is shown in Figure 1.

### 3.2 Characteristics of included studies

**FIGURE 2:**
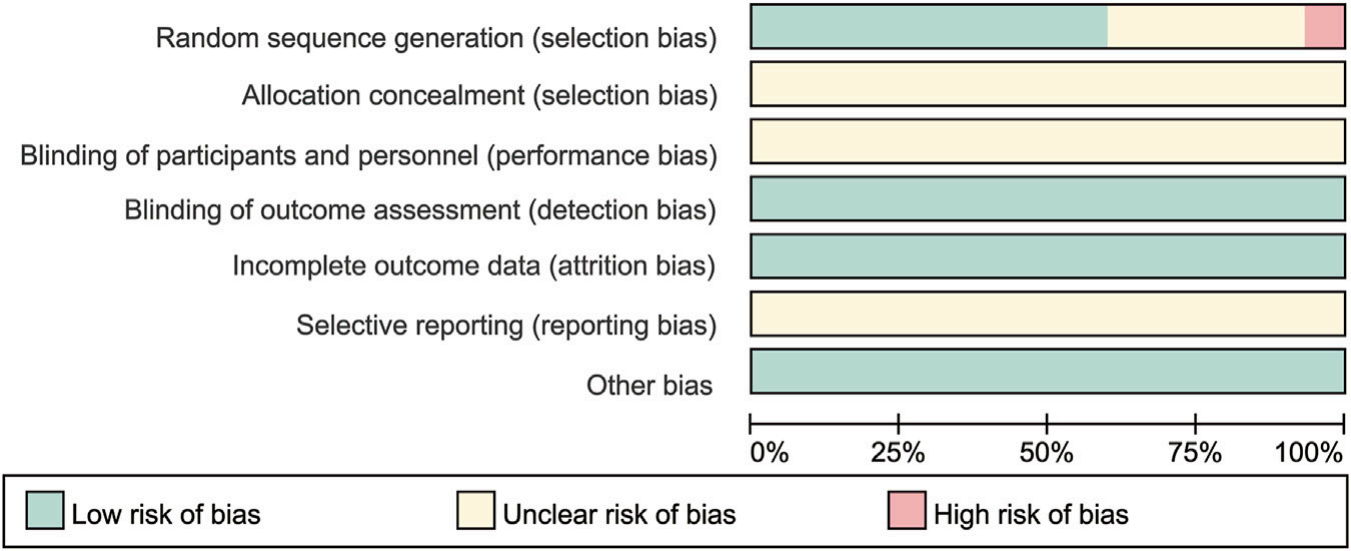
Risk of bias graph.

Among the 30 RCTs, GC + TWM, reported in 12 RCTs involving 577 cases, was used most frequently, followed by GC + CAA reported in 8 RCTs involving 275 cases, CNI + TWM reported in 6 RCTs involving 206 cases, TWM reported in 6 RCTs involving 398 cases, GC + CNI reported in 6 RCTs involving 270 cases, CNI reported in 5 RCTs involving 144 cases, GC + CNI + TWM reported in 4 RCTs involving 161 cases, GC + MMF/LEF + TWM reported in 4 RCTs involving 108 cases, GC reported in 4 RCTs involving 93 cases, GC + MMF reported in 2 RCTs involving 66 cases. GC + CAA + CNI + TWM, GC + CNI + MMF + TWM, and CAA + MMF + TWM were only mentioned in 1 RCT, respectively, involving 38 cases, 32 cases, and 42 cases. Detailed information on outcome measures was reported in all 30 included studies. As all the included studies were Chinese RCTs, baseline information was comparable between the control and trial groups (Table 1). All included RCTs were of medium-to high-quality, as shown in Figure 2. As only six articles among the included studies reported the level of the anti-phospholipase A2 receptor (anti-PLA2R) antibody, we can only narratively summarize the results of these six studies. The detailed results are shown in the Supplementary Table S3. In addition, the TWMs used in all the included studies are active ingredients extracted from the Euonymus plant *Tripterygium wilfordii* Hook. f. [Celastraceae]. The manufacturer, national drug approval number, Ingredient, administration method and other relevant information of TWM used in all included studies are provided in Supplementary Table S1 according to the ConPhyMP statement (Heinrich et al., 2022).

**TABLE 1:** Key features of included studies.

Author	Region	Population	Age (mean, SD)	Gender (female, male)	Scr(μmol/L)	Albumin (g/L)	24 h-UTP (g/d)	Comparisons	Outcomes
Ai and Wang. (2022)	China	408	52.41, 6.33	183, 225	89.78, 8.91	22.03, 3.16	6.07, 1.39	GC + TWM	TR, 24 h-UTP, AEs
								TWM	
Chen et al. (2022)	China	97	56.52, 5.19	35, 62	131.55, 26.23	22.11, 3.57	8.04, 1.16	GC + CNI + TWM	TR, AEs
								GC + CNI	
Wang. (2021)	China	50	52.00, 2.97	31, 19	NR	23.00, 3.96	5.05, 1.04	GC + TWM	TR, 24 h-UTP, AEs
								GC	
Ding. (2021)	China	90	36.40, 5.88	28, 62	106.25, 19.21	26.70, 7.47	5.25, 0.17	GC + CNI + TWM	TR
								GC + CNI	
Wang. (2020)	China	76	60.50, 7.50	31, 45	70.53, 11.59	23.05, 4.70	7.39, 1.41	GC + CNI + TWM	TR, AEs
								GC + CNI	
Zhang et al. (2020)	China	127	47.77, 5.08	59, 68	78.05, 10.40	19.72, 5.32	5.21, 1.48	CNI + TWM	TR, AEs
								GC + CNI	
Xiong and Huang. (2020)	China	64	33.31, 2.58	19, 45	NR	13.64, 0.67	3.58, 1.63	CNI + TWM	TR, AEs
								CNI	
Zhou et al. (2020)	China	76	49.33, 2.37	27, 49	NR	25.55, 5.10	5.87, 0.71	GC + CAA + CNI + TWM	TR, 24 h-UTP, AEs
								GC + CAA	
Bao. (2020)	China	108	48.97, 5.78	50, 58	NR	14.35, 2.18	3.14, 1.16	GC + TWM	TR, 24 h-UTP, AEs
								TWM	
Liang et al. (2020)	China	92	61.46, 5.18	38, 54	84.60, 8.34	13.27, 1.34	3.46, 0.39	CNI + TWM	TR
								CNI	
Feng. (2020)	China	52	40.72, 5.81	17, 35	164.19, 15.02	NR	6.98, 2.27	CNI + TWM	TR, AEs
								CNI	
Zhu et al. (2020)	China	50	47.93, 3.20	21, 29	127.76, 21.22	20.94, 2.95	5.46, 1.42	GC + TWM	TR, 24 h-UTP, AEs
								GC	
Mo et al. (2019)	China	42	50.00, 9.52	19, 23	97.84, 21.36	20.66, 3.65	7.19, 3.24	GC + MMF/LEF + TWM GC + MMF	TR, 24 h-UTP, AEs
Xue et al. (2019)	China	91	51.09, 8.76	33, 58	93.43, 70.71	15.10, 9.88	9.38, 4.99	GC + TWM	TR, 24 h-UTP
								TWM	
Liu et al. (2018)	China	60	44.65, 11.98	19,41	70.50, 18.62	19.91, 6.25	5.70, 2.57	GC + CNI + MMF + TWM GC + CAA	TR, 24 h-UTP, AEs
Yu et al. (2018)	China	68	37.50, 15.60	27, 41	85.85, 22.13	21.01, 4.83	5.48, 1.28	TWM	24 h-UTP, AEs
								GC + CAA	
Gong et al. (2018)	China	84	55.70, 10.39	30, 54	94.25, 44.03	23.00, 5.12	7.65, 3.48	GC + CAA	TR, 24 h-UTP, AEs
								CAA + MMF + TWM	
Guo et al. (2017)	China	84	35.30, 3.08	18, 66	143.81, 24.79	NR	7.11, 2.21	GC + TWM	TR, 24 h-UTP, AEs
								GC + CAA	
Wang. (2017)	China	40	33.95, 5.76	23, 17	NR	NR	NR	CNI + TWM	TR, AEs
								CNI	
Zhang. (2017)	China	50	NR	21, 29	103.77, 23.86	26.65, 5.75	5.26, 2.11	GC + MMF/LEF + TWM	TR, 24 h-UTP
								TWM	
Wang et al. (2016)	China	41	46.06, 6.75	18, 23	77.53, 1.36	17.10, 2.49	NR	GC + TWM	TR, AEs
								GC + CAA	
Yang et al. (2015)	China	90	45.25, 13.27	37, 53	123.85, 13.99	2.84, 1.27	5.77, 1.33	GC + MMF/LEF + TWM GC + MMF	TR, 24 h-UTP, AEs
Peng et al. (2015)	China	40	29.75, 12.10	23,17	92.05, 18.25	13.35, 4.32	3.52, 1.22	CNI + TWM	TR, AEs
								CNI	
Zuo et al. (2014)	China	100	43.08, 11.96	35, 65	68.07, 11.40	24.55, 3.17	6.04, 2.30	GC + TWM	TR, AEs
								GC + CNI	
Qiu et al. (2014)	China	58	53.40, 16.39	18, 40	85.75, 29.30	20.95, 7.04	6.75, 3.29	GC + CNI + TWM GC + CNI	TR, AEs
Ma et al. (2014)	China	60	NR	28, 32	NR	18.95, 6.06	5.35, 0.89	GC + TWM	TR, 24 h-UTP
								GC	
Feng et al. (2011)	China	33	NR	8, 25	103.91, 23.91	27.18, 5.79	5.21, 2.17	GC + MMF/LEF + TWM GC + CAA	TR, 24 h-UTP, AEs
Liu et al. (2009)	China	84	44.45, 11.86	23, 61	-	25.66, 4.61	5.85, 2.40	GC + TWM	TR, AEs
								TWM	
Sun and He. (2012)	China	90	41.98, 16.36	27, 63	72.68, 19.25	23.72, 5.75	5.32, 2.25	GC + TWM	TR, AEs
								GC + CAA	
Xie and Xu. (2021)	China	26	50.70, 10.69	9, 17	91.00, 11.14	22.80, 8.66	2.55, 2.01	GC + TWM	TR, 24 h-UTP
								GC	

Abbreviation: TR, total remission; 24 h-UTP, 24-h urine total protein; AEs, adverse events; Scr, serum creatinine; NR, not reported; TWM, tripterygium wilfordii multiglycosides; CNI, calcineurin inhibitor; GC, glucocorticoids; CAA, alkylating agents; MMF, mycophenolate mofetil; LEF, leflunomide.

### 3.3 Evidence network

**FIGURE 3:**
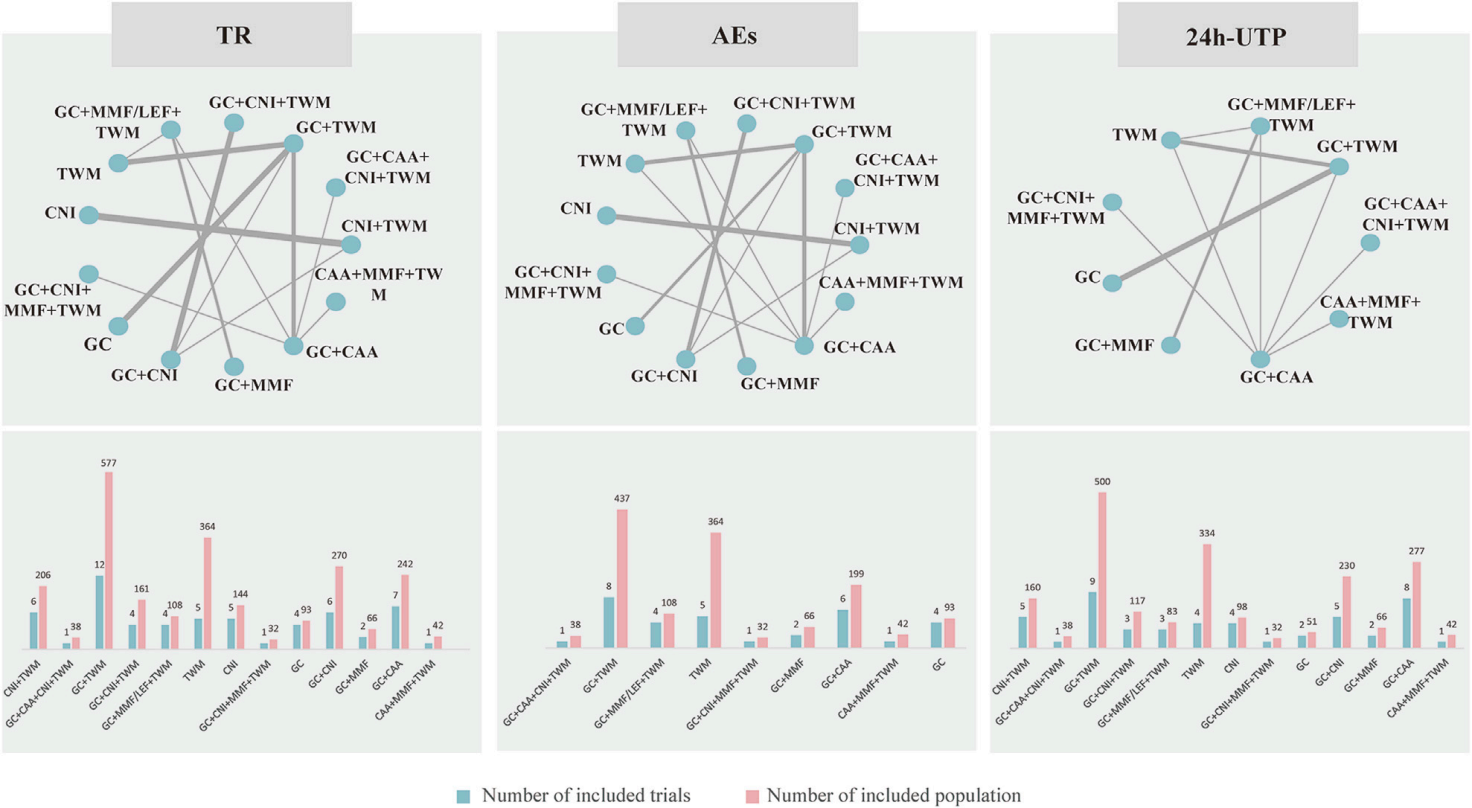
Netplot of TR, 24 h-UTP and AEs. Abbreviation: TR, total remission; 24 h-UTP, 24-h urine total protein; AEs, adverse events; CNI, calcineurin inhibitor; GC, glucocorticoids; TWM, tripterygium wilfordii multiglycosides; CAA, alkylating agents; MMF, mycophenolate mofetil; LEF, leflunomide. Notes: The thickness of the line represents the number of trials included in the relevant comparison.

The evidence network is shown in Figure 3; each node indicates an intervention plan, and the thickness of the connecting line between nodes indicates the number of included studies under that intervention. The analysis of TR and AE involved 13 interventions, respectively; the analysis of 24H-UTP involved 9 interventions. The number of studies reporting GC + TWM was the largest (12 RCTs).

### 3.4 Convergence and inconsistency tests

The potential scale reduction parameter (PSRF) values for all outcome measures were close to 1, indicating a good convergence of this NMA algorithm. Therefore, the consistency model was adopted for analysis, and the results were reliable. As shown in Supplementary Files S2, S3, S4, our analysis of all outcome measures was tested for inconsistency by using the node-splitting method, and all *p*-values were greater than 0.05. Direct comparison and indirect comparison were consistent in results, with no statistical difference. This indicated that our results were highly consistent and reliable. The heterogeneity analysis of the TR and the incidence of AEs (Supplementary File S2, S3) showed a small overall heterogeneity, and thus a fixed-effects model was chosen for NMA. In contrast, significant heterogeneity was found when the 24 h-UTP of the groups was compared, and thus we chose a random-effects model to perform NMA.

### 3.5 Results of NMA

#### 3.5.1 TR

**FIGURE 4:**
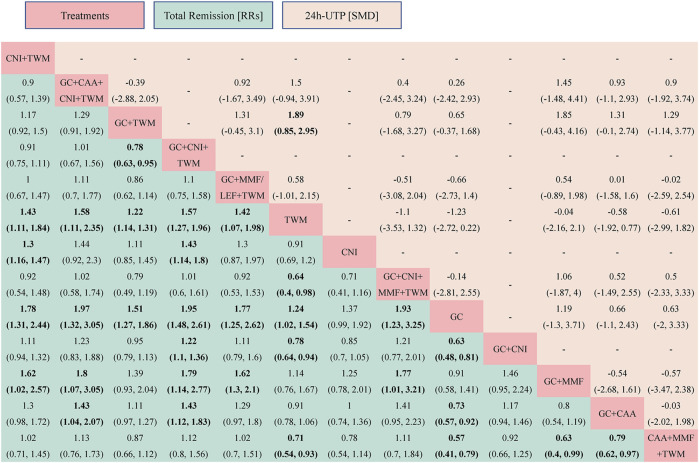
The total remission and the 24 h-UTP were analyzed by network meta-analysis. Major achievements are expressed in bold. Abbreviation: TR, total remission; 24 h-UTP, 24-h urine total protein; TWM, tripterygium wilfordii multiglycosides; CNI, calcineurin inhibitor; GC, glucocorticoids; CAA, alkylating agents; MMF, mycophenolate mofetil; LEF, leflunomide; RRs, risk ratios; SMD, standard mean difference.

TR was reported in 29 studies including 13 interventions and 2,343 patients. The evidence network is shown in Figure 3. When compared with that of the GC group, The RR [95% CI] for each intervention of the following group were: CNI + TWM (1.80 [1.30, 2.40]), GC + CAA + CNI + TWM (2.00 [1.30, 3.10]), GC + TWM (1.50 [1.30, 1.90]), GC + CNI + TWM (2.00 [1.50, 2.60]), GC + MMF/LEF + TWM (1.80 [1.30, 2.60]), TWM (1.20 [1.00, 1.50]), CNI (1.40 [0.99,1.90]), GC + CNI + MMF + TWM (1.90 [1.20,3.20]), GC + CNI (1.60 [1.20, 2.10]), GC + MMF (1.10 [0.71,1.70]), GC + CAA (1.40 [1.10,1.80]), and CAA + MMF + TWM (1.70 [1.30,2.50]). The league table showed significant differences in the TR between interventions. According to the probability ranking plot and SUCRA values, the GC + CNI + TWM group had the highest TR (SUCRA value of 85.58%), followed by GC + CAA + CNI + TWM (81.50%), GC + CNI + MMF + TWM (78.02%), CNI + TWM (72.13%), GC + MMF/LEF + TWM (70.50%), CAA + MMF + TWM (68.28%), GC + CNI (54.80%), GC + TWM (46.23%), CNI (30.61%), GC + CAA (29.70%), TWM (17.72%), and GC + MMF (11.63%), while the GC group had the lowest TR (3.29%) (Figure 4; Table 2, and Supplementary File S2). Among all the plans, the addition and subtraction plan of CNI + TWM was the most effective in the treatment of IMN.

**TABLE 2:** SUCRA of total remission, 24-h urine total protein and the adverse events.

	SUCRA		
Treatments	TR (%)	24h-UTP(%)	AEs (%)
CNI + TWM	72.13	-	79.96
GC + CAA + CNI + TWM	81.50	71.57	30.34
GC + TWM	46.23	88.12	72.82
GC + CNI + TWM	85.58	-	75.87
GC + MMF/LEF + TWM	70.50	42.94	39.70
TWM	17.72	18.06	49.23
CNI	30.61	-	76.38
GC + CNI + MMF + TWM	78.02	59.24	58.94
GC	3.29	64.08	27.04
GC + CNI	54.80	-	51.71
GC + MMF	11.63	23.47	56.50
GC + CAA	29.70	39.88	15.96
CAA + MMF + TWM	68.28	42.65	15.54

Abbreviation: TR, total remission; 24 h-UTP, 24-h urine total protein; AEs, adverse events; TWM, tripterygium wilfordii multiglycosides; CNI, calcineurin inhibitor; GC, glucocorticoids; CAA, alkylating agents; MMF, mycophenolate mofetil; LEF, leflunomide; SUCRA, surface under the cumulative ranking area.

**FIGURE 5:**
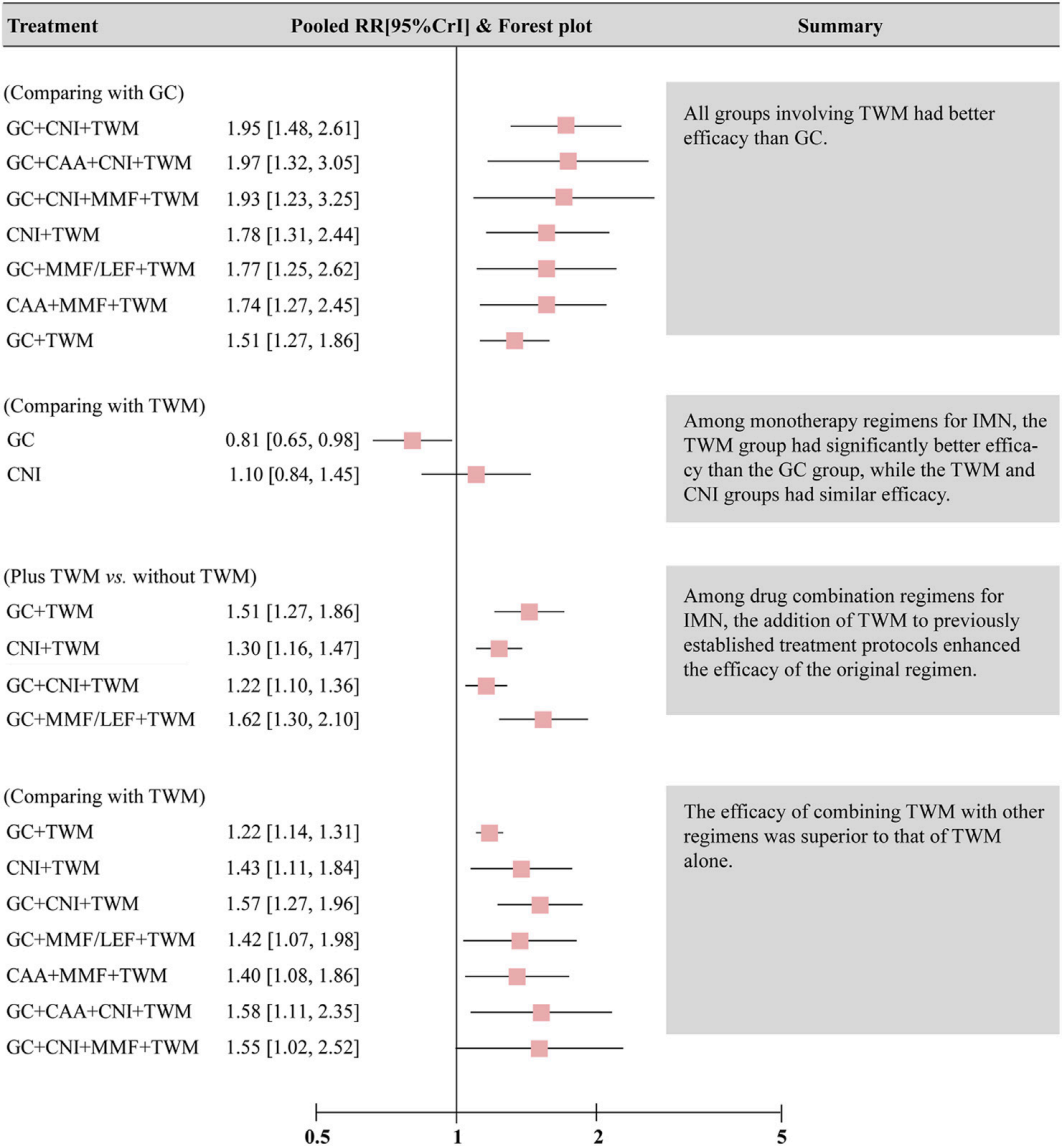
Summary of results of network meta-analysis of total remission. Abbreviation: CrI, credit interval, CNI, calcineurin inhibitor; GC, glucocorticoids; TWM, tripterygium wilfordii multiglycosides; CAA, alkylating agents; MMF, mycophenolate mofetil; LEF, leflunomide.

The results also showed that: ① Except that CNI and GC + MMF had similar efficacy to GC, the TR of other plans was superior to GC. In other words, all groups containing TWM had better efficacy than GC; ② In the treatment for IMN with single drug, the efficacy of TWM was clearly better than that of GC, while the efficacy of TWM and CNI was similar; ③ In terms of the drug combination plans for IMN, the addition of TWM to a previous mature plan could enhance its efficacy; ④ TWM combined with other drugs showed better efficacy than TWM alone (Figure 5).

#### 3.5.2 24 h-UTP quantification

A total of 16 studies with 9 interventions and 1,379 individuals were included in the NMA of 24 h-UTP (Figure 3). The PSRF value for NMA was 1, indicating a high degree of convergence. Although the league table showed no significant differences among the interventions in reducing 24 h-UTP, it can be concluded that GC + TWM had the best efficacy in reducing 24 h-UTP; TWM had the worst efficacy, and GC + TWM was superior to the TWM according to probability ranking plots and SUCRA values.

The results of NMA showed that there was no significant difference between all intervention measures and GC group (Figure 4; Supplementary File S2).

In contrast to the TR test results, GC + TWM was the most effective in reducing 24 h-UTP (SUCRA value of 88.12%), followed by GC + CAA + CNI + TWM (71.57%), GC (64.08%), GC + CNI + MMF + TWM (59.24%), GC + MMF/LEF + TWM (42.94%), CAA + MMF + TWM (42.65%), GC + CAA (39.88%), and GC + MMF (23.47%). TWM was the least effective in reducing 24 h-UTP (18.06%) (Table 2; Supplementary File S3).

#### 3.5.3 AEs

A total of 24 studies with 13 interventions and 2,028 individuals were included in the NMA of the incidence of AEs (Figure 3). No serious AEs occurred in all included studies. The most common adverse reactions in this study were elevated transaminase and digestive tract symptoms such as nausea and vomiting. The details are shown in Supplementary Table S2. However, such symptoms occurred only in an individual case in all studies and there was no difference between groups. Therefore, we did not analyze different adverse events separately, but analyzed the incidence of overall adverse events.

The PSRF value for NMA was 1, indicating a good convergence. Although the league table showed no significant differences in AEs among interventions, it can be concluded that CNI + TWM was the best and CAA + MMF + TWM was the worst among these interventions in terms of the incidence of AEs according to probability ranking plots and SUCRA values. When the efficacy was similar between the groups, the GC + CNI + TWM group had a lower incidence of AEs than the GC + CAA group, and the GC + TWM group had a lower incidence of AEs than the GC + CAA and CAA + MMF + TWM groups. Therefore, as the efficacy of these medication plans was equivalent, the plan with fewer AEs was recommended.

**FIGURE 6:**
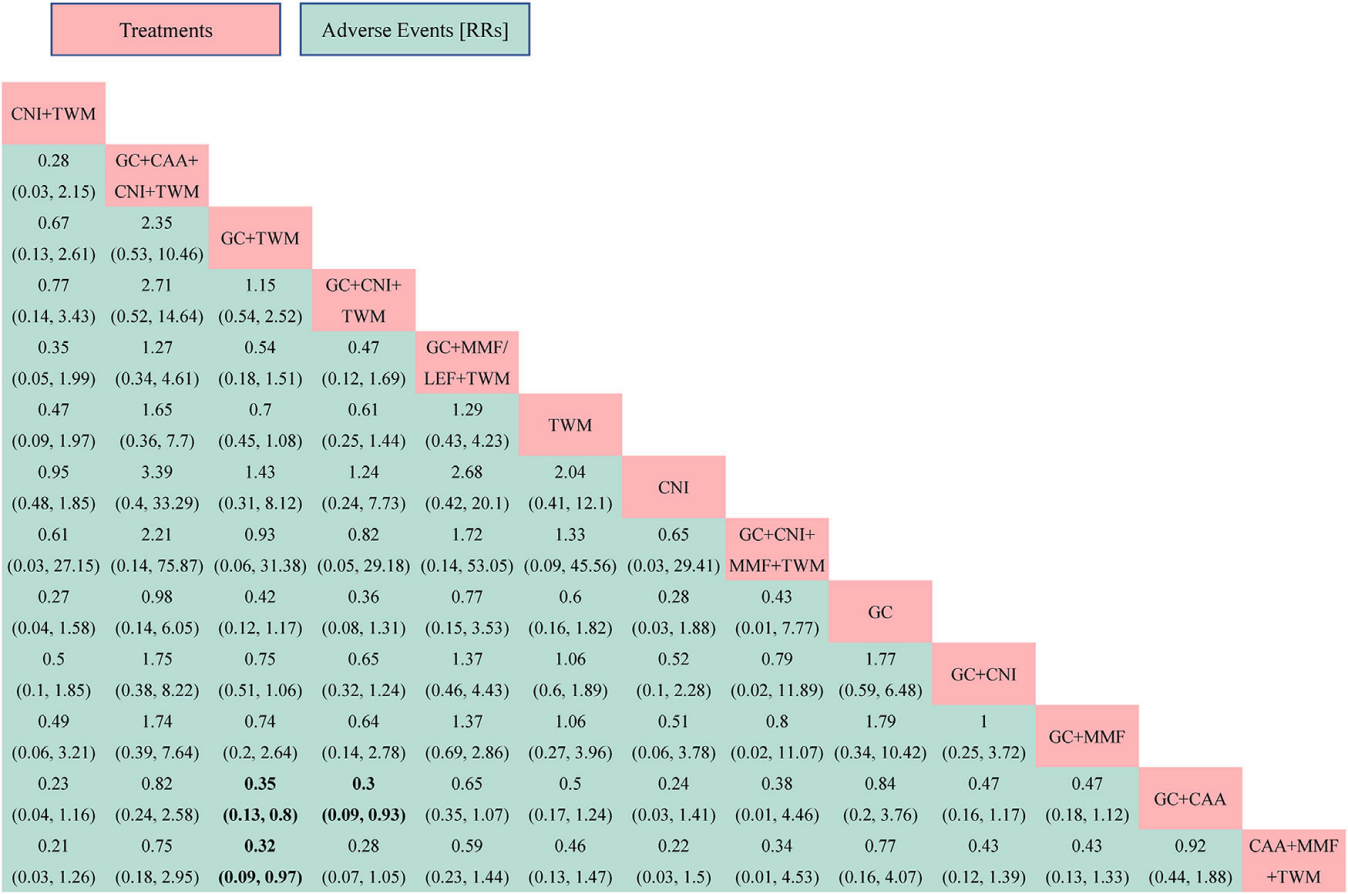
The adverse events was analyzed by network meta-analysis. Major achievements are expressed in bold. Abbreviation: AEs, adverse events; TWM, tripterygium wilfordii multiglycosides; CNI, calcineurin inhibitor; GC, glucocorticoids; CAA, alkylating agents; MMF, mycophenolate mofetil; LEF, leflunomide; RRs, risk ratios.

The analysis showed that there was no significant difference between all intervention measures and GC (Figure 6; Supplementary File S4).

According to probability ranking plots and SUCRA values, the CNI + TWM group ranked lowest in the incidence of an AE (SUCRA value of 79.96%), followed by CNI (76.38%), GC + CNI + TWM (75.87%), GC + TWM (72.82%), GC + CNI + MMF + TWM (58.94%), GC + MMF (56.50%), GC + CNI (51.71%), TWM (49.23%), GC + MMF/LEF + TWM (39.70%), GC + CAA + CNI + TWM (30.34%), GC (27.04%), and GC + CAA (15.96%). CAA + MMF + TWM was the highest in the incidence of an AE (15.54%) (Table 2; Supplementary File S4).

## 4 Sensitivity analysis

For the results of TR, 24 h-UTP, and AEs, sensitivity analysis showed that there was no huge change in the overall effect when any study was excluded, indicating that our results were robust and reliable. In addition, sensitivity analysis was also performed according to the risk level of progression of renal impairment in patients with IMN. The results showed that all the included studies were medium-high risk except for 5 low-risk articles. Hence, we removed these 5 low-risk articles and re-performed the network meta-analysis. The results showed that the total effective rate and 24 h-UTP results we before obtained were still stable (Supplementary Files S1, S2).

## 5 Discussion

In this study, we found that the addition of TWM may be beneficial to patients with IMN. It may enhance the efficacy of previously established treatment protocols without leading to additional safety risks.

For a long time, GC and immunosuppressive drugs have been the main drugs in the treatment of IMN in modern medicine. However, no satisfactory efficacy has been achieved in either single or combined use (Perna et al., 2004). A series of AEs caused by the long-term use of immunosuppressive drugs have been a problem troubling patients, such as serious infections, abnormal blood glucose, and impaired liver function. Besides, the high price, easy relapse, and failure to improve the long-term survival of patients are additional major problems in immunosuppressive treatment (Liu et al., 2019). TCM has shown some advantages in treating IMN. TWM is relatively positively effective in reducing urinary protein and protecting renal function (Chen et al., 2010; Ma et al., 2015). Its main mechanisms of action include (Gong et al., 2018): ① anti-inflammatory: inhibit the release of inflammatory cytokines and inflammatory mediators, such as TGF-β1, TNF-α, IL-2, and IL-6, to reduce the inflammatory response of renal tissues (Wu et al., 2017; Chang et al., 2018; Wang, 2018). ② Immunosuppression: suppress cellular and humoral immunity, and attenuate cell-mediated and immune complex-mediated renal injury. ③ Protect podocyte cytoskeletal proteins: It has been reported that TPL alleviates proteinuria and podocyte injury in IMN rats with no obvious AEs and is protective against podocyte injury induced by complement C5b-9 membrane attack complex *in vitro*. Wan et al. also observed that TPL at 0–4 mg/L was not toxic to podocytes (Wan et al., 2020). It can regulate PI3K/Akt/mTOR, MAPK and other signaling pathways to help reduce puromycin aminonucleoside (PAN)-induced damage to the actin cytoskeleton and microfilament-associated synaptic proteins, thus maintaining normal function of podocytes and glomerular filtration membrane, repairing podocyte damage, and producing major anti-proteinuric effects (Zheng et al., 2008; Chen et al., 2010; Zhang et al., 2022). ④ Inhibit the proliferation of thylakoid cells and extracellular matrix. TPL can protect the kidney by mitigating epithelial mesenchymal transition (EMT) of hexokinase (HK-2) cells through PI3K/Akt signaling pathway (Xue et al., 2018) and inhibiting Akt/mTOR signaling pathway, thus inhibiting the proliferation of glomerular tethered cells in diabetic nephropathy (Han et al., 2017). ⑤ Protect the glomerular basement membrane: TWM can reduce the deposition of antigen-antibody immune complex in the glomerular basement membrane, improve the permeability of the glomerular basement membrane, and repair the glomerular charge barrier; ⑥ Protect the damaged renal tubulointerstitium: it can repair the damaged renal tubules and alleviate renal interstitial fibrosis, etc (Ma et al., 2015; Li et al., 2017). Therefore, TWM, as an immunosuppressant, has a good protective effect on the kidney and can be widely promoted and used at home and abroad.

In this study, we found that the addition and subtraction plan of CNI + TWM was the most effective among all the plans for IMN, and the GC + CNI + TWM group had the highest TR, while the GC group had the lowest TR. The top two plans in terms of TR were GC + CNI + TWM and GC + CAA + CNI + TWM. GC is a drug with immunosuppressive effects (Olejniczak et al., 2022). GC alone has poor efficacy, but GC in combination with CTX or CNI can be used as an initial therapy plan for most IMN patients, with a remission of 70%–80% (Radhakrishnan and Cattran, 2012). If monotherapy is considered, KDIGO recommended CNI as the best alternative to the initial plan because CNI can reduce 24 h-UTP in patients with IMN to a greater extent (Beck et al., 2013). The *Clinical Practice Guidelines for IMN with Traditional Chinese Medicine* (2021) pointed out that for IMN patients with a low risk of progression of renal impairment, conservative treatment without RAS inhibitors and oral administration of TWM monotherapy for 3–12 months can achieve better CR and TR (Yang et al., 2023). Thus, it suggests that TWM alone promotes the prognosis of IMN patients, and adding TWM to the two effective intervention plans, GC + CNI and GC + CAA + CNI, improves the TR of previous therapy plans. Therefore, we recommend the appropriate addition of TWM to drug combination plans in order to achieve better efficacy of previously established therapy plans.

In this study, we also found that TR of the TWM group was much better than that of the GC group among monotherapy plans for IMN. This suggests that TWM can be an alternative option for patients with contraindications to GC or poor results of GC treatment. Also, the present study found similar TR between the TWM and CNI groups for the treatment of IMN, suggesting that further studies are needed to compare the efficacy and safety of TWM and CNI in the future. In addition, a therapy in combination with immunosuppressive drugs is recommended for IMN patients with persistent proteinuria or deteriorating renal function (Hofstra et al., 2013). Through this study, we found that the addition of TWM to a previously established therapy plan for IMN would improve the efficacy of the original plan without additional safety risks. For example, considering the poor efficacy of GC alone, Feng et al. added TWM to the GC plan to treat patients with IMN and found that the TR was 87.5% after 6 months, noticeably higher than 54.17% in the GC group (Feng et al., 2015). The representative drugs of CNI are mainly TAC and CsA, and studies have shown that the PR and CR of TAC + TWM for IMN after 10 months were 1.57 and 2.69 times higher than those of TAC alone (Shang et al., 2018). Peng et al. (Peng et al., 2015)observed that TAC + TWM was more effective than TAC in reducing 24hUTP. The addition of TWM also resulted in smoother blood levels in IMN patients, and thus substantially reduced the risk of disease relapse. It has also been reported that GC + CNI therapy remains the classic plan for the treatment of IMN until today, and the addition of TWM also has marked advantages in optimizing the TR of this plan (Gao, 2013; Du et al., 2019). Jinyan Cui et al. (Cui and Li, 2020) also found that the GC + TAC + TWM plan had a TR of 93.3% for the treatment of IMN after 6 months, which was evidently higher than that of 70.0% for patients in the GC + TAC group, and the safety of the two groups was similar. These findings are consistent with the conclusion that GC + CNI + TWM was more effective than GC + CNI in the present study. MMF has a low level of evidence for the treatment of IMN due to its short application time frame (Tran et al., 2015). According to the literature (Mo et al., 2019), compared to the GC + MMF plan, the GC + MMF + TWM plan for IMN had an increased TR by 28.58% and also more advantages in improving patients’ 24 h-UTP and serum albumin levels. In addition, this study found that the combination of TWM and other drugs was more effective than TWM alone, regardless of TWM as a variable, so combination plans are recommended in order to maximize the benefits to patients. Liu et al. (Liu et al., 2009) noted that the TR of the GC + TWM plan for IMN for 12 months was 32.8% higher than that of TWM alone, and their efficacy ranking was as follows: GC + TWM > TWM > GC (Liu et al., 2009; Feng et al., 2015), which provided a theoretical reference for clinical medication plans. Two meta-analyses also showed a larger advantage of CNI + TWM in reducing 24 h-UTP in IMN patients compared with TWM alone (Zheng et al., 2019; Zhou et al., 2021).

Furthermore, this study found that the plans with the top four TRs were all based on the addition and subtraction plan of CNI + TWM. Therefore, from the perspective of effectiveness and economic benefits, it is recommended to choose CNI + TWM therapy plan with less drug use in clinical practice. Meanwhile, the TR of GC + TWM group was similar to that of GC + MMF/LEF + TWM group, GC + CAA + CNI + TWM group and GC + CNI + MMF + TWM group, so we recommend GC + TWM therapy plan with less drug use in clinical practice. In addition to the effectiveness and safety considerations of a medication plan, the actual cost of medication should also be taken into account. In China, TWM has its place in the treatment of autoimmune diseases by virtue of its excellent immunosuppressive effects and few side effects. TWM is widely accepted among massive patients for its cost-effectiveness and affordability (priced at 1/6 and 1/9 of CsA and TAC) (Jin et al., 2020). Incremental cost-effectiveness analysis by Xu Wanyi et al. (Xu et al., 2021) showed that CAA and CsA were more affordable compared with MMF and TAC. It is crucial to develop an individualized therapy plan based on the patient’s disease progression and economic situation. Therefore, given efficacy, safety, and economic benefits, clinical investigators are recommended to choose their medication plans according to the actual drug prices in their respective countries and regions when plans are similar with only minor differences in drugs. For example,: As CNI + TWM is equivalent in efficacy to GC + CNI, the drug prices of TWM and GC are referenced; As GC + TWM is as effective as GC + TWM and GC + MMF, the prices of TWM and MMF are involved; As GC + TWM and GC + CAA have a similar efficacy, the prices of TWM and CAA are drawn on; as TWM and CNI are the same in efficacy, and the prices of them are considered.

The selection of immunosuppressive therapy plans for IMN has been controversial due to its varying natural course and prognosis. In this study, for the first time, we systematically evaluated the efficacy of 13 TWM-involved intervention plans for IMN by the NMA. In recent years, the efficacy and safety of immunosuppressants in patients with IMN have been reported (Ren et al., 2017), but our study is the only systematic review and meta-analysis that focuses on TWM, which was our major advantage. The results provide preliminary evidence that the application of TWM may be beneficial to patients with membranous nephropathy. Its addition may further enhance the efficacy of a previously established therapy plan without additional safety risks, providing clinicians with ideas and rationale for clinical prescriptions. However, despite the rigorous statistical analysis, this study has the following limitations: 1. The reliability of the results may be affected by the varying durations of follow-up and inconsistent doses in the included studies; 2. The presence of spontaneous remission in adults with IMN may interfere with the determination of the TR; 3. The included studies of some interventions were small in its quantity and sample size, which may lead to “false positives” in comparisons and ranking, and the possible unpublished negative results may also affect the stability of the study results; 4. The RCTs included in this study were mainly from Chinese literature, which may be related to the fact that the adoption of proprietary Chinese medicines is mainly limited to China, and there is language and geographical bias to some extent. However, from the perspective of evidence-based medicine, TWM may be relatively effective for Chinese adults with IMN. Because it has not been widely used worldwide, the results of this study cannot demonstrate whether racial differences affect the efficacy of therapy plans; 5. Due to the limitation of the included studies, the studies focused on the immediate efficacy, and there was a lack of data on the long-term efficacy such as recurrence rate and 3-year survival rate. NMA was not conducted to evaluate the relative advantages and disadvantages. It is recommended that future studies focus more on long-term efficacy indicators that reflect the quality of life of patients with membranous nephropathy; 6. The subjects in the original literature include young and old patients, and there may be some differences in response to treatment among patients of different age groups; 7. Some included RCTs did not clearly report their randomization, allocation concealment, blinding and pre-registration of the research plan, which may lead to a bias. Future RCTs focusing on this area need to be performed or reported more formally to improve the reliability of these findings; 8. Many manufacturers produce TWM with different ingredient contents in the market. As such manufacturers may have different manufacturing processes, their TWMs may vary in ingredient contents, and thus in the actual efficacy. However, the included studies did not provide the drug content information of each manufacturer in more detail, which may compromise the credibility of this study; 9. For IMN, the index of anti-PLA2R antibody is very important. However, due to the fact that only six articles in our included studies have reported this data, we could not perform meta-analysis of this index. More studies on this index are warranted in the future. 10. This study tried to analyze the remission of patients with medium-high- and low-risk IMN using TWM respectively. However, in the actual analysis process, we found that most of the included studies were conducted in patients with medium- and high-risk IMN, and only 5 studies were conducted in patients with low-risk IMN. The number of articles was small, so we could not distinguish the remission of patients with medium-high- and low-risk IMN using TWM. More studies are desired to report low-risk renal impairment progression in IMN patients in the future. Due to the limited number and quality of the included studies, the above findings need to be validated by more high-quality studies in order to provide better evidence to support clinical prescriptions. Based on the limitations of the existing studies, our findings need to be carefully applied.

## 6 Conclusion

In summary, the current evidence suggests that reasonable immunosuppressive therapy should be started as soon as possible for ESRD-prone IMN patients with no spontaneous remission, in addition to symptomatic supportive therapy. The application of the herbal immunosuppressant TWM may be beneficial to patients with IMN. Its addition may enhance the efficacy of previously established treatment protocols and does not lead to additional safety risks. In particular, GC + CNI + TWM, GC + TWM, and CNI + TWM can be the preferred therapy plans for IMN in clinical decision-making because of their better efficacy and high safety.

## Data Availability

The original contributions presented in the study are included in the article/Supplementary Material, further inquiries can be directed to the corresponding author.

## Abbreviations

IMN, idiopathic membranous nephropathy; TR, total remission; CR, complete remission; PR, partial remission; 24 h-UTP, 24-h urine total protein; uPCR, urinary protein creatinine ratio; Scr, serum creatinine; AEs, adverse events; NMA, network meta-analysis; RCTs, randomized controlled trials; TWMs, Tripterygium wilfordii multiglycosides; TPL, triptolide; CNI, calcineurin inhibitor; CsA, cyclosporine; TAC, tacrolimus; GC, glucocorticoids; CAA, alkylating agents; CTX, cyclophosphamide; MMF, mycophenolate mofetil; LEF, leflunomide; CON, non-immunosuppressive therapies; RRs, risk ratios; CIs, Confidence intervals; SMD, standard mean difference; PSRF, potential scale reduction factors; SUCRA, surface under the cumulative ranking area; ESRD, end-stage renal disease.
