# Supplementary material for: Efficacy and safety of Tripterygium wilfordii multiglucoside for idiopathic membranous nephropathy: a systematic review with bayesian meta-analysis
Source: Front Pharmacol. 2023 Aug 2;14:1183499. doi: 10.3389/fphar.2023.1183499 (PMC10442163; doi:10.3389/fphar.2023.1183499)
Supplement: Supplementary file 1 [file DataSheet1.docx]

**Abbreviation:** TR, total remission; 24h-UTP, 24-hour urine total protein; AEs, adverse events; TWM, tripterygium wilfordii multiglycosides; CNI, calcineurin inhibitor; GC, glucocorticoids; CAA, alkylating agents; MMF, mycophenolate mofetil; LEF, leflunomide; NR, not reported; 1, CNI+TWM; 2, GC+CAA+CNI+TWM; 3, GC+TWM; 4, GC+CNI+TWM; 5, GC+MMF/LEF+TWM; 6, TWM; 7, CNI; 8, GC+CNI+MMF+TWM; 9, GC; 11, GC+CNI; 12, GC+MMF; 13, GC+CAA; 15, CAA+MMF+TWM.

**S1 Literature Searching Process**

(‘extramembranous glomerulonephritis’ OR ‘Extramembranous Glomerulopathy’ OR ‘Glomerulonephritides, Idiopathic Membranous’ OR ‘Glomerulonephritides, Membranous’ OR ‘glomerulonephritis, extramembranous’ OR ‘Glomerulonephritis, Idiopathic Membranous’ OR ‘Glomerulonephritis, Membranous’ OR ‘Glomerulonephropathy, Membranous’ OR ‘Glomerulopathy, Extramembranous’ OR ‘Glomerulopathy, Membranous’ OR ‘Heymann Nephritis’ OR ‘Idiopathic Membranous Glomerulonephritides’ OR ‘Idiopathic Membranous Glomerulonephritis’ OR ‘Idiopathic Membranous Nephropathy’ OR ‘membranous glomerulitis’ OR ‘Membranous Glomerulonephritides’ OR ‘Membranous Glomerulonephritides, Idiopathic’ OR ‘Membranous Glomerulonephritis’ OR ‘Membranous Glomerulonephritis, Idiopathic’ OR ‘Membranous Glomerulonephropathy’ OR ‘Membranous Glomerulopathy’ OR ‘membranous nephritis’ OR ‘Membranous Nephropathy’ OR ‘Membranous Nephropathy, Idiopathic’ OR ‘Nephritis, Heymann’ OR ‘nephritis, membranous’ OR ‘Nephropathy, Idiopathic Membranous’ OR ‘Nephropathy, Membranous’):ti,ab,kw

(‘hypoglaucums, Tripterygium’ OR ‘lei gong teng’ OR ‘Leigong Teng’ OR ‘Leigong Tengs’ OR ‘leigongteng’ OR ‘Teng, Leigong’ OR ‘Tengs, Leigong’ OR ‘thunder god vine’ OR ‘Thundergod Vine’ OR ‘Thundergod Vines’ OR ‘Tripterygium’ OR ‘Tripterygium wilfordii’ OR ‘Tripterygium hypoglaucum’ OR ‘Tripterygium hypoglaucums’ OR ‘Tripterygium wilfordius’ OR ‘Tripterygiums’ OR ‘Vine, Thundergod’ OR ‘Vines, Thundergod’ OR ‘wilfordius, Tripterygium’):ti,ab,kw

**S2**

**2.1 Total remission (Result of network meta-analysis for total remission)**


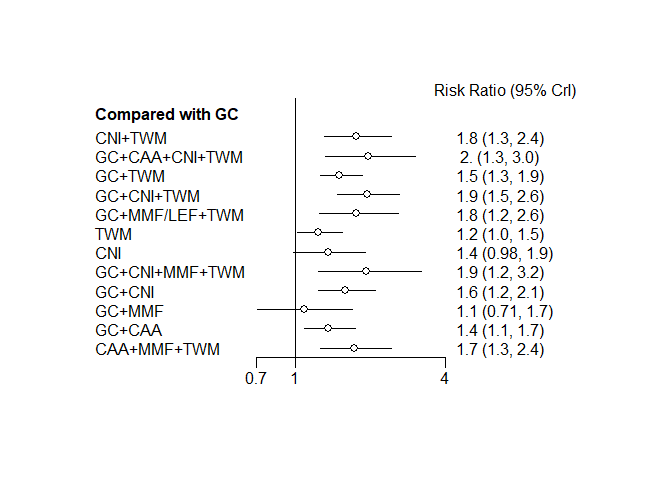


**2.2 Total remission (Probability ranking plots)**


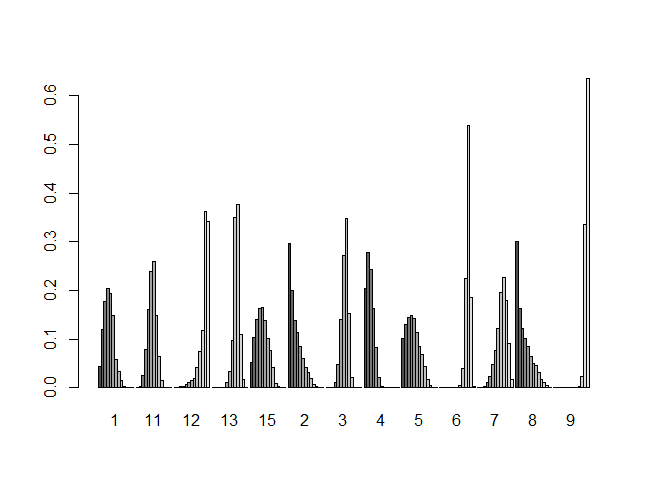


**2.3**

2.3.1 Total remission (Inconsistency tests)


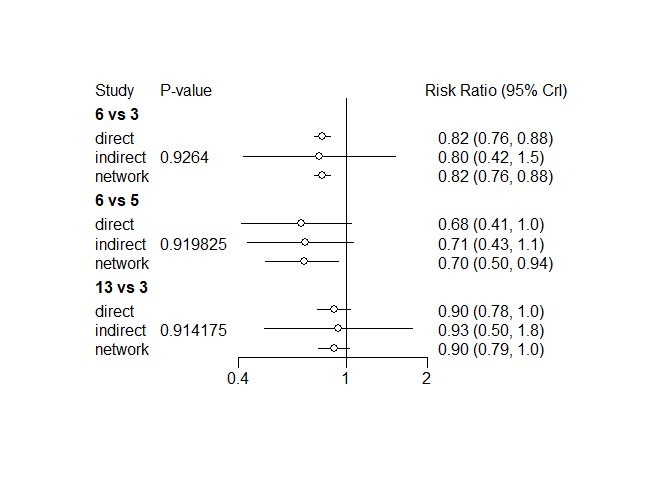


2.3.2 Total remission (Inconsistency tests)


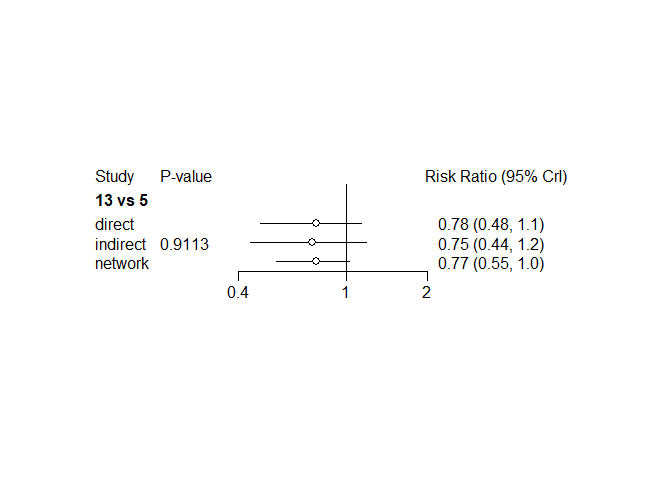


**2.4**

2.4.1 Total remission (Analysis of heterogeneity)


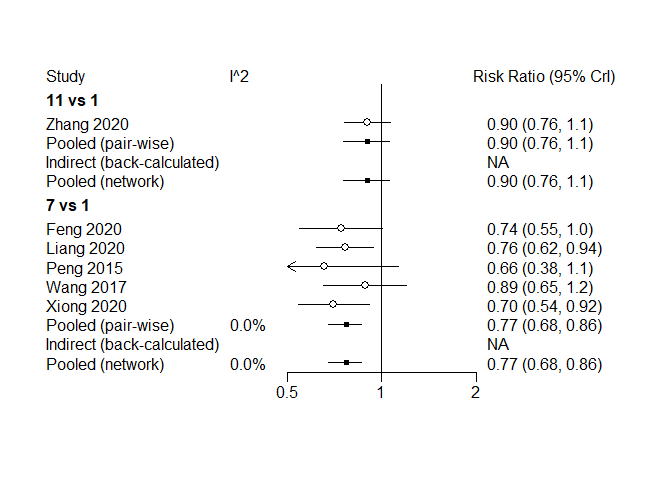


2.4.2 Total remission (Analysis of heterogeneity)


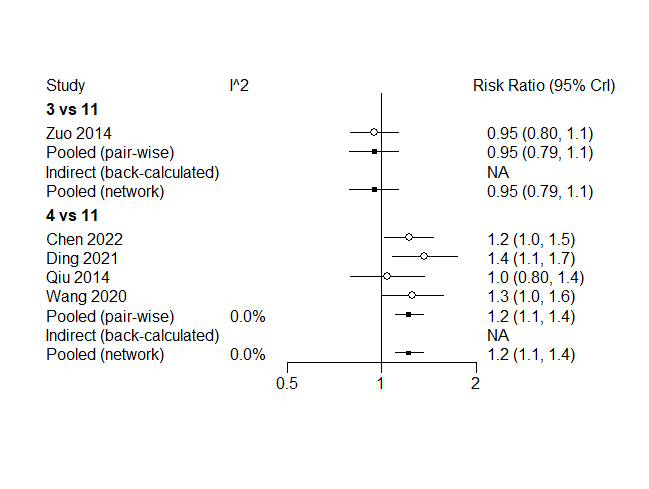


2.4.3 Total remission (Analysis of heterogeneity)


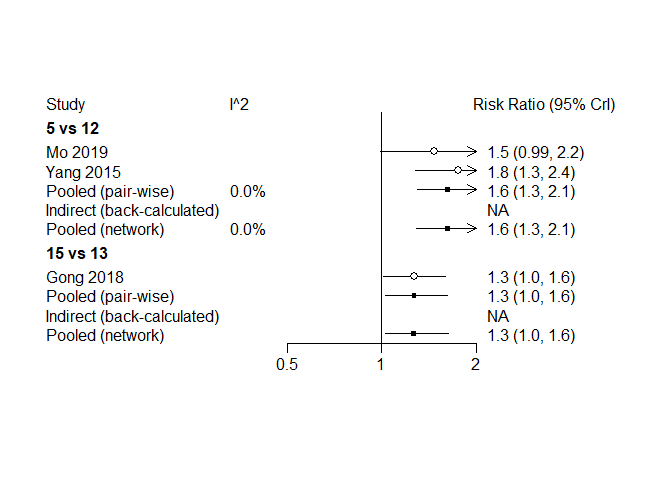


2.4.4 Total remission (Analysis of heterogeneity)


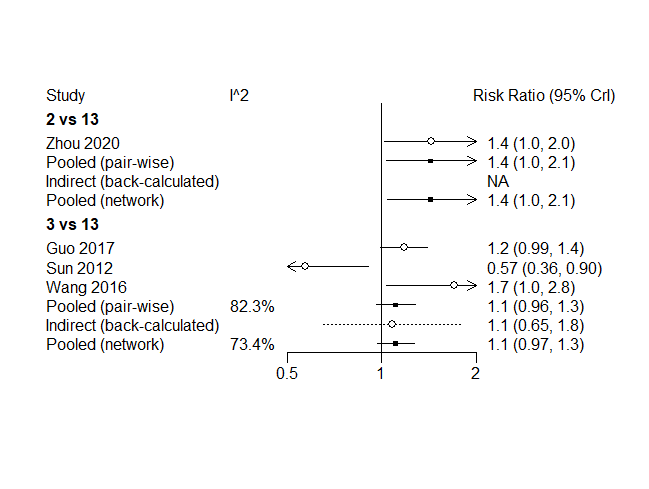


2.4.5 Total remission (Analysis of heterogeneity)


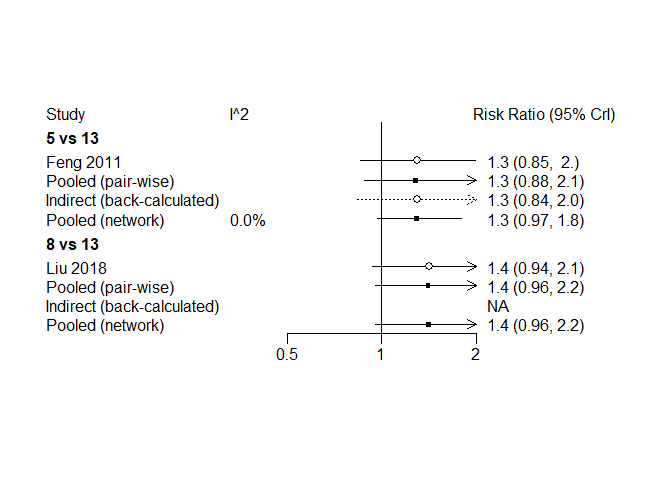


2.4.6 Total remission (Analysis of heterogeneity)


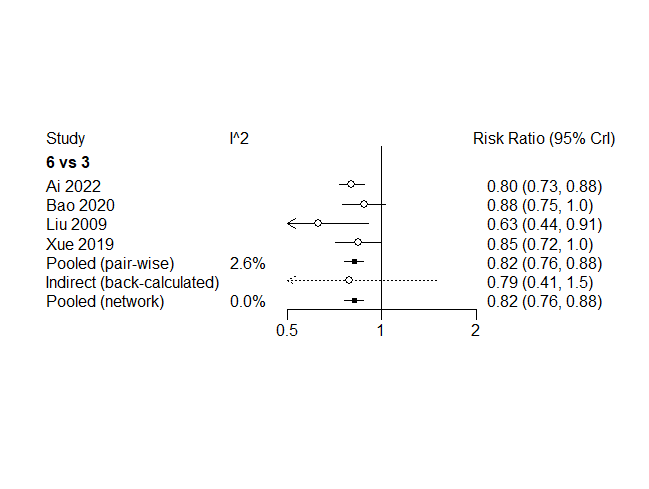


2.4.7 Total remission (Analysis of heterogeneity)


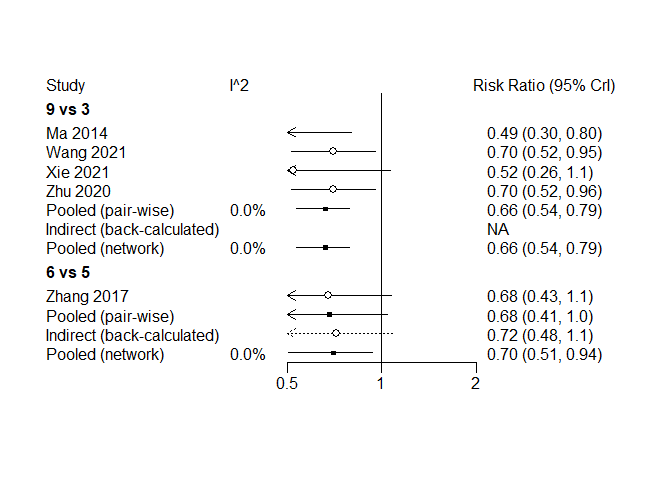


**2.****5 Sensitivity analysis of total remission was also performed according to the risk level of progression of renal impairment in patients with IMN.**

2.5.1 Total remission (Result of network meta-analysis for total remission)


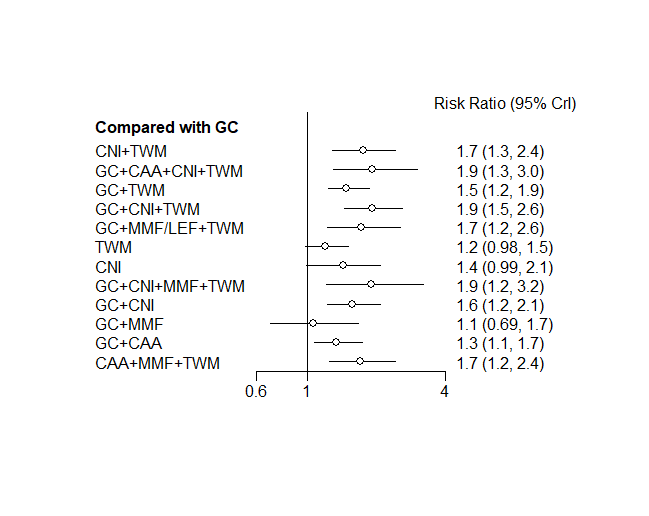


2.5.2 Total remission (Probability ranking plots)


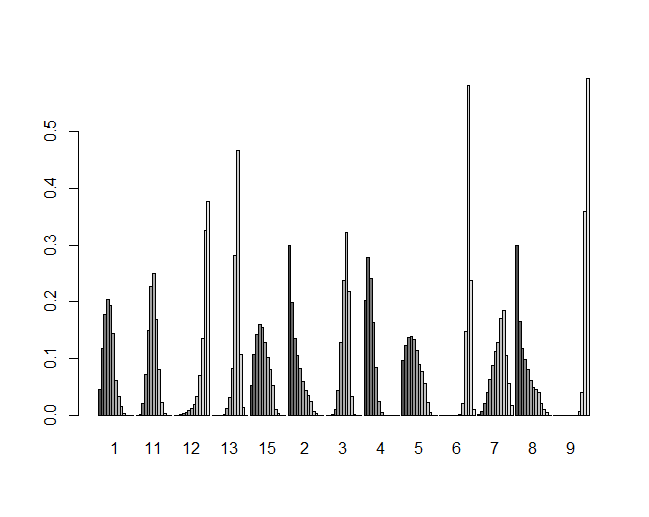


2.5.3 Total remission (The total remission was analyzed by network meta-analysis.)

| **CNI+TWM** | 1.11  (0.72, 1.74) | 0.86  (0.67, 1.09) | 1.1  (0.9, 1.34) | 0.99  (0.67, 1.49) | 0.69  (0.53, 0.88) | 0.82  (0.66, 0.99) | 1.09  (0.67, 1.84) | 0.57  (0.41, 0.78) | 0.9  (0.76, 1.06) | 0.61  (0.39, 0.97) | 0.77  (0.58, 1.02) | 0.98  (0.69, 1.42) |
| --- | --- | --- | --- | --- | --- | --- | --- | --- | --- | --- | --- | --- |
| 0.9  (0.57, 1.39) | **GC+CAA+**  **CNI+TWM** | 0.77  (0.52, 1.1) | 0.99  (0.64, 1.49) | 0.89  (0.56, 1.42) | 0.62  (0.42, 0.89) | 0.74  (0.45, 1.19) | 0.98  (0.58, 1.71) | 0.52  (0.33, 0.77) | 0.81  (0.53, 1.21) | 0.55  (0.33, 0.93) | 0.7  (0.48, 0.96) | 0.89  (0.58, 1.32) |
| 1.17  (0.92, 1.5) | 1.29  (0.91, 1.91) | **GC+TWM** | 1.28  (1.05, 1.59) | 1.15  (0.87, 1.62) | 0.81  (0.74, 0.87) | 0.96  (0.69, 1.32) | 1.27  (0.84, 2.03) | 0.67  (0.54, 0.81) | 1.05  (0.89, 1.26) | 0.71  (0.48, 1.07) | 0.9  (0.79, 1.04) | 1.14  (0.89, 1.52) |
| 0.91  (0.75, 1.11) | 1.01  (0.67, 1.56) | 0.78  (0.63, 0.95) | **GC+CNI+**  **TWM** | 0.9  (0.63, 1.33) | 0.63  (0.5, 0.78) | 0.75  (0.55, 0.99) | 0.99  (0.62, 1.65) | 0.52  (0.39, 0.69) | 0.82  (0.73, 0.91) | 0.56  (0.36, 0.87) | 0.7  (0.55, 0.9) | 0.89  (0.64, 1.26) |
| 1.01  (0.67, 1.48) | 1.12  (0.7, 1.78) | 0.87  (0.62, 1.15) | 1.11  (0.75, 1.58) | **GC+MMF/**  **LEF+TWM** | 0.7  (0.5, 0.93) | 0.83  (0.52, 1.27) | 1.1  (0.66, 1.86) | 0.58  (0.39, 0.82) | 0.91  (0.63, 1.28) | 0.62  (0.48, 0.77) | 0.78  (0.56, 1.04) | 0.99  (0.67, 1.44) |
| 1.45  (1.13, 1.89) | 1.61  (1.12, 2.4) | 1.24  (1.15, 1.35) | 1.6  (1.29, 2) | 1.44  (1.08, 2.01) | **TWM** | 1.19  (0.85, 1.65) | 1.58  (1.04, 2.54) | 0.83  (0.66, 1.02) | 1.31  (1.08, 1.59) | 0.89  (0.6, 1.32) | 1.12  (0.96, 1.31) | 1.42  (1.09, 1.9) |
| 1.22  (1.01, 1.53) | 1.35  (0.84, 2.24) | 1.04  (0.76, 1.45) | 1.34  (1.01, 1.8) | 1.21  (0.78, 1.92) | 0.84  (0.61, 1.17) | **CNI** | 1.33  (0.79, 2.34) | 0.7  (0.48, 1.02) | 1.1  (0.85, 1.45) | 0.75  (0.45, 1.25) | 0.94  (0.67, 1.34) | 1.2  (0.8, 1.83) |
| 0.92  (0.54, 1.49) | 1.02  (0.59, 1.74) | 0.79  (0.49, 1.19) | 1.01  (0.61, 1.61) | 0.91  (0.54, 1.52) | 0.63  (0.39, 0.96) | 0.75  (0.43, 1.27) | **GC+CNI+**  **MMF+TWM** | 0.53  (0.32, 0.83) | 0.83  (0.5, 1.3) | 0.56  (0.31, 0.98) | 0.71  (0.45, 1.05) | 0.9  (0.55, 1.42) |
| 1.74  (1.29, 2.43) | 1.93  (1.29, 3.01) | 1.49  (1.24, 1.85) | 1.91  (1.45, 2.58) | 1.73  (1.22, 2.56) | 1.2  (0.98, 1.51) | 1.43  (0.98, 2.09) | 1.9  (1.2, 3.16) | **GC** | 1.57  (1.22, 2.08) | 1.07  (0.69, 1.68) | 1.34  (1.07, 1.74) | 1.71  (1.24, 2.43) |
| 1.11  (0.94, 1.32) | 1.23  (0.83, 1.88) | 0.95  (0.79, 1.13) | 1.22  (1.1, 1.36) | 1.1  (0.78, 1.6) | 0.76  (0.63, 0.92) | 0.91  (0.69, 1.18) | 1.21  (0.77, 1.99) | 0.64  (0.48, 0.82) | **GC+CNI** | 0.68  (0.44, 1.05) | 0.86  (0.68, 1.07) | 1.09  (0.8, 1.51) |
| 1.64  (1.03, 2.58) | 1.81  (1.08, 3.07) | 1.4  (0.94, 2.06) | 1.8  (1.15, 2.78) | 1.62  (1.3, 2.1) | 1.13  (0.76, 1.66) | 1.34  (0.8, 2.21) | 1.78  (1.02, 3.2) | 0.94  (0.6, 1.45) | 1.47  (0.96, 2.25) | **GC+MMF** | 1.26  (0.85, 1.85) | 1.6  (1.02, 2.52) |
| 1.29  (0.98, 1.72) | 1.43  (1.04, 2.07) | 1.11  (0.96, 1.27) | 1.42  (1.11, 1.83) | 1.28  (0.97, 1.79) | 0.89  (0.76, 1.04) | 1.06  (0.75, 1.5) | 1.41  (0.95, 2.21) | 0.74  (0.58, 0.94) | 1.17  (0.93, 1.46) | 0.79  (0.54, 1.18) | **GC+CAA** | 1.27  (1.03, 1.63) |
| 1.02  (0.71, 1.46) | 1.13  (0.76, 1.73) | 0.87  (0.66, 1.13) | 1.12  (0.8, 1.56) | 1.01  (0.69, 1.49) | 0.7  (0.53, 0.92) | 0.84  (0.55, 1.26) | 1.11  (0.7, 1.83) | 0.59  (0.41, 0.81) | 0.92  (0.66, 1.26) | 0.62  (0.4, 0.98) | 0.79  (0.61, 0.97) | **CAA+MMF**  **+TWM** |

2.5.4 Total remission (SUCRA of total remission and the 24-hour urine total protein)

| Treatments | SUCRA | |
| --- | --- | --- |
|  | TR | 24h-UTP |
| CNI+TWM | 72.07% | - |
| GC+CAA+CNI+TWM | 81.26% | 71.61% |
| GC+TWM | 44.95% | 92.10% |
| GC+CNI+TWM | 85.46% | - |
| GC+MMF/LEF+TWM | 68.86% | 39.54% |
| TWM | 16.16% | 13.45% |
| CNI | 38.63% | - |
| GC+CNI+MMF+TWM | 77.85% | 59.17% |
| GC | 3.88% | 70.04% |
| GC+CNI | 53.52% | - |
| GC+MMF | 10.86% | 20.59% |
| GC+CAA | 28.90% | 40.76% |
| CAA+MMF+TWM | 67.59% | 42.75% |

**S3**

**3.1 24-hour urine total protein (Result of network meta-analysis for 24h-UTP)**


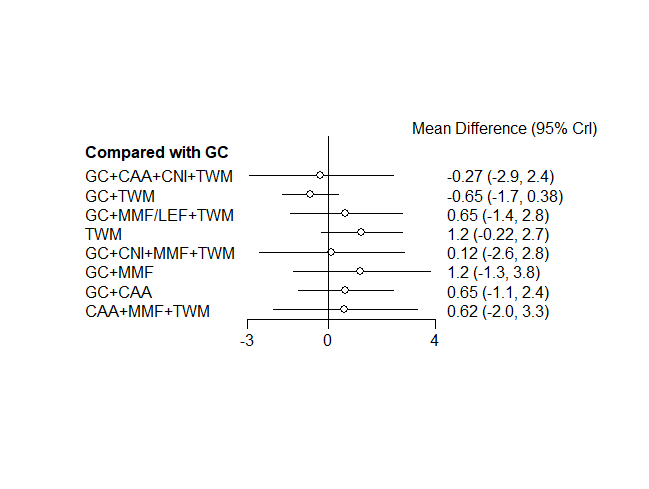


**3.2 24-hour urine total protein (Probability ranking plots)**


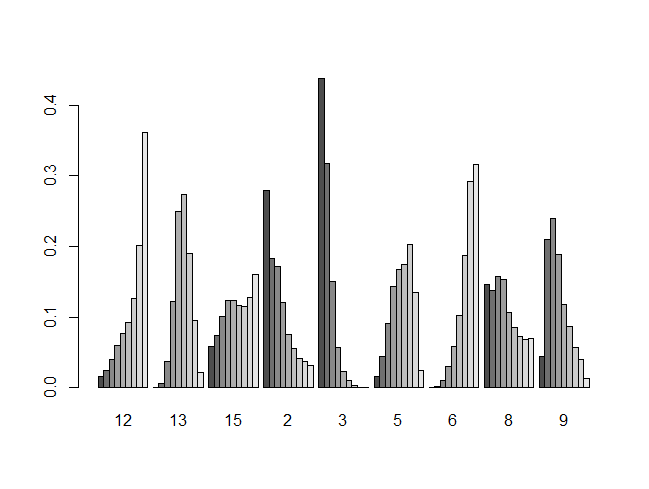


**3.3**

3.3.1 24-hour urine total protein (Inconsistency tests)


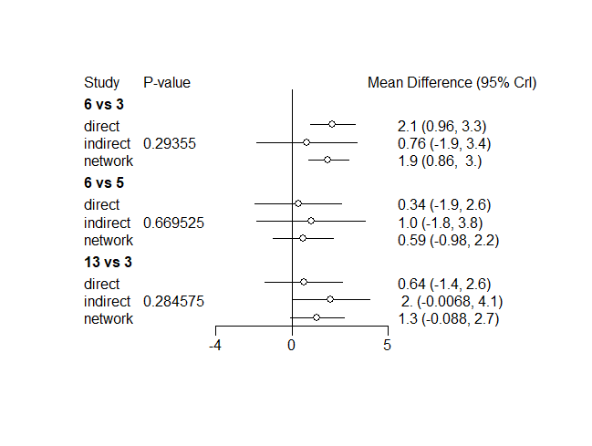


3.3.2 24-hour urine total protein (Inconsistency tests)


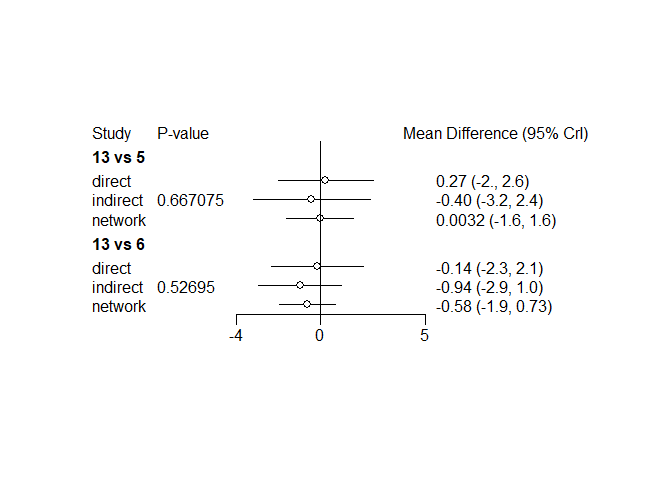


**3.4**

3.4.1 24-hour urine total protein (Analysis of heterogeneity)


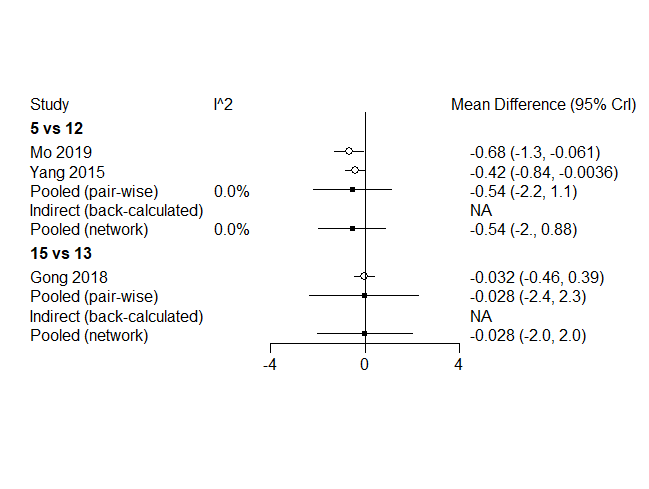


3.4.2 24-hour urine total protein (Analysis of heterogeneity)


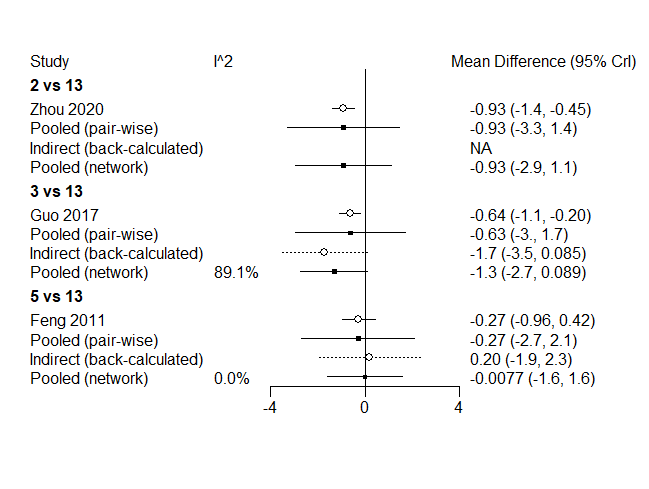


3.4.3 24-hour urine total protein (Analysis of heterogeneity)


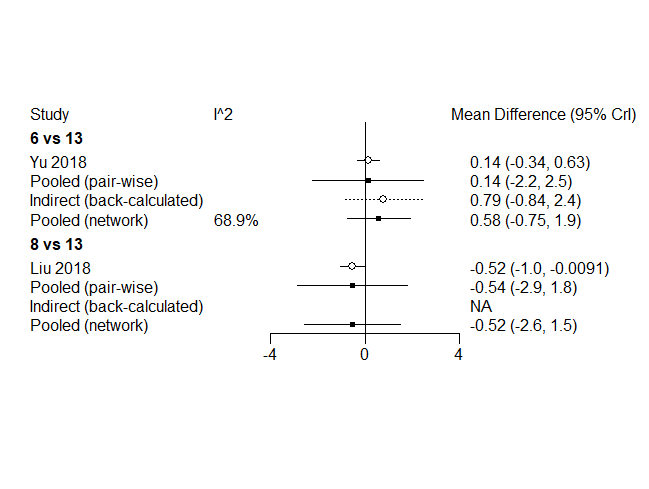


3.4.4 24-hour urine total protein (Analysis of heterogeneity)


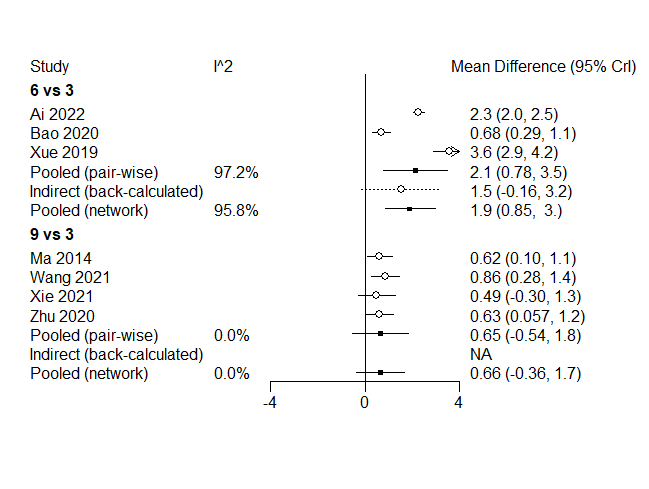


3.4.5 24-hour urine total protein (Analysis of heterogeneity)


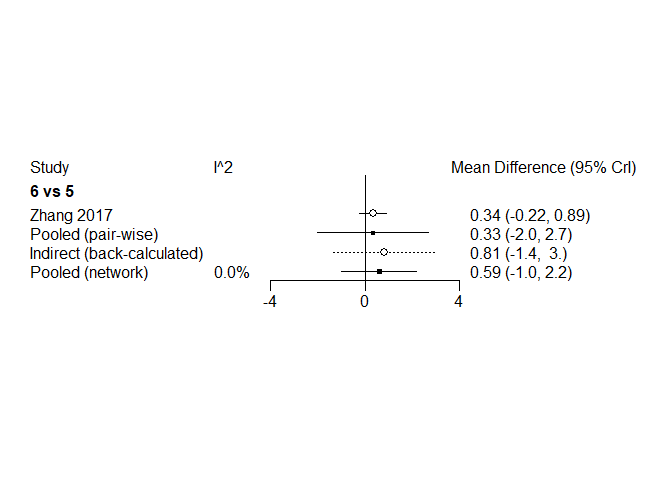


**3.5 Sensitivity analysis of 24-hour urine total protein was also performed according to the risk level of progression of renal impairment in patients with IMN.**

3.5.1 24-hour urine total protein (Result of network meta-analysis for total remission)


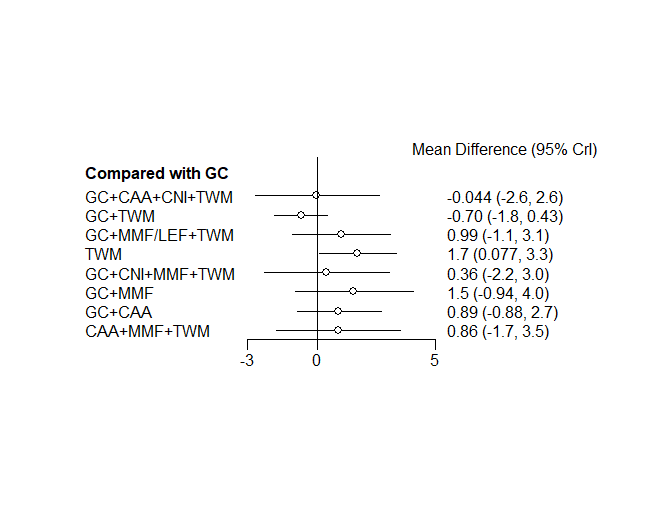


3.5.2 24-hour urine total protein (Probability ranking plots)


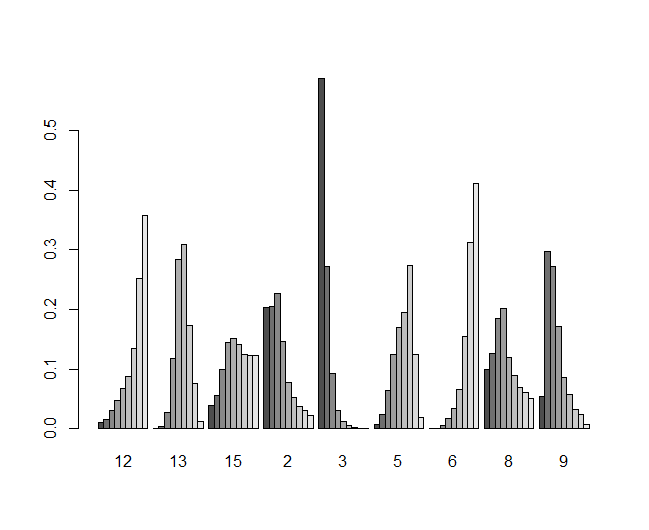


3.5.3 24-hour urine total protein (The 24-hour urine total protein was analyzed by network meta-analysis.)

| **GC+CAA+**  **CNI+TWM** | -0.66  (-3.07, 1.68) | 1.03  (-1.43, 3.47) | 1.71  (-0.61, 4.00) | 0.40  (-2.33, 3.14) | 0.04  (-2.61, 2.62) | 1.56  (-1.25, 4.40) | 0.92  (-0.98, 2.84) | 0.89  (-1.80, 3.61) |
| --- | --- | --- | --- | --- | --- | --- | --- | --- |
| 0.66  (-1.68, 3.07) | **GC+TWM** | 1.69  (-0.07, 3.48) | 2.37  (1.20, 3.59) | 1.06  (-1.31, 3.50) | 0.70  (-0.43, 1.83) | 2.23  (0.00, 4.49) | 1.59  (0.20, 3.01) | 1.56  (-0.80, 3.97) |
| -1.03  (-3.47, 1.43) | -1.69  (-3.48, 0.07) | **GC+MMF/LEF**  **+TWM** | 0.68  (-0.83, 2.23) | -0.63  (-3.08, 1.87) | -0.99  (-3.09, 1.09) | 0.54  (-0.82, 1.92) | -0.10  (-1.62, 1.44) | -0.14  (-2.56, 2.32) |
| -1.71  (-4.00, 0.61) | -2.37  (-3.59, -1.20) | -0.68  (-2.23, 0.83) | **TWM** | -1.31  (-3.63, 1.03) | -1.67  (-3.34, -0.08) | -0.14  (-2.21, 1.92) | -0.78  (-2.10, 0.53) | -0.81  (-3.12, 1.51) |
| -0.40  (-3.14, 2.33) | -1.06  (-3.50, 1.31) | 0.63  (-1.87, 3.08) | 1.31  (-1.03, 3.63) | **GC+CNI+**  **MMF+TWM** | -0.36  (-3.02, 2.25) | 1.17  (-1.69, 3.99) | 0.52  (-1.42, 2.46) | 0.49  (-2.23, 3.20) |
| -0.04  (-2.62, 2.61) | -0.70  (-1.83, 0.43) | 0.99  (-1.09, 3.09) | 1.67  (0.08, 3.34) | 0.36  (-2.25, 3.02) | **GC** | 1.53  (-0.94, 4.05) | 0.89  (-0.88, 2.71) | 0.86  (-1.74, 3.49) |
| -1.56  (-4.40, 1.25) | -2.23  (-4.49, 0.00) | -0.54  (-1.92, 0.82) | 0.14  (-1.92, 2.21) | -1.17  (-3.99, 1.69) | -1.53  (-4.05, 0.94) | **GC+MMF** | -0.64  (-2.69, 1.43) | -0.68  (-3.48, 2.13) |
| -0.92  (-2.84, 0.98) | -1.59  (-3.01, -0.20) | 0.10  (-1.44, 1.62) | 0.78  (-0.53, 2.10) | -0.52  (-2.46, 1.42) | -0.89  (-2.71, 0.88) | 0.64  (-1.43, 2.69) | **GC+CAA** | -0.03  (-1.93, 1.87) |
| -0.89  (-3.61, 1.80) | -1.56  (-3.97, 0.80) | 0.14  (-2.32, 2.56) | 0.81  (-1.51, 3.12) | -0.49  (-3.20, 2.23) | -0.86  (-3.49, 1.74) | 0.68  (-2.13, 3.48) | 0.03  (-1.87, 1.93) | **CAA+MMF+**  **TWM** |

**S4**

**4.1 Adverse events (Result of network meta-analysis for adverse events)**


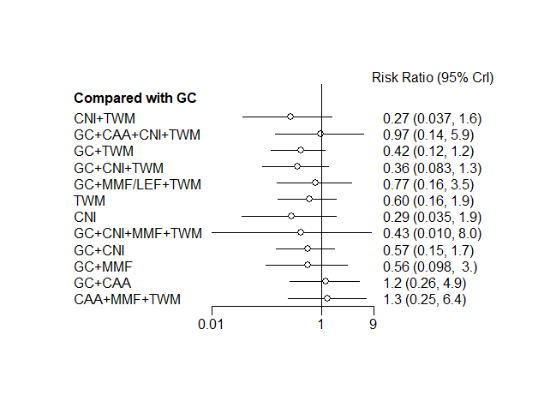


**4.2 Adverse events (Probability ranking plots)**


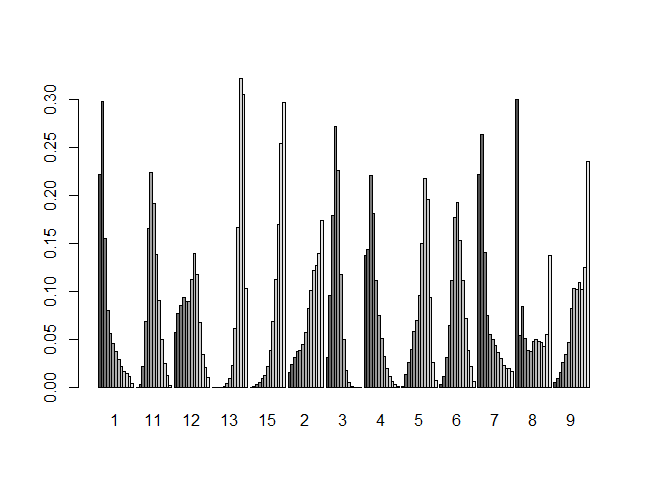


**4.3 Adverse events (Inconsistency tests)**


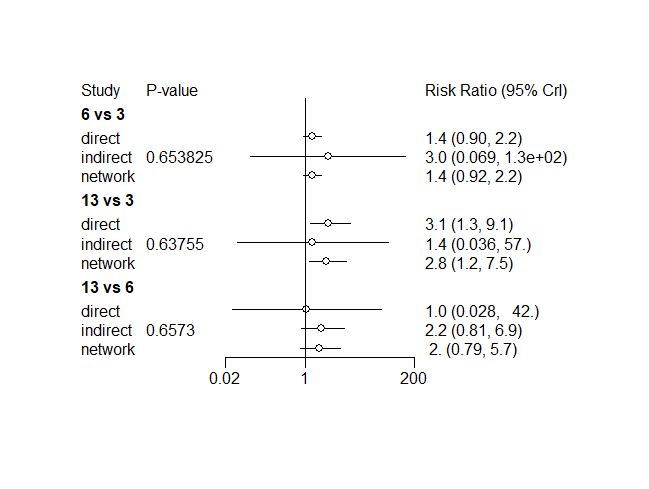


**4.4**

4.4.1 Adverse events (Analysis of heterogeneity)


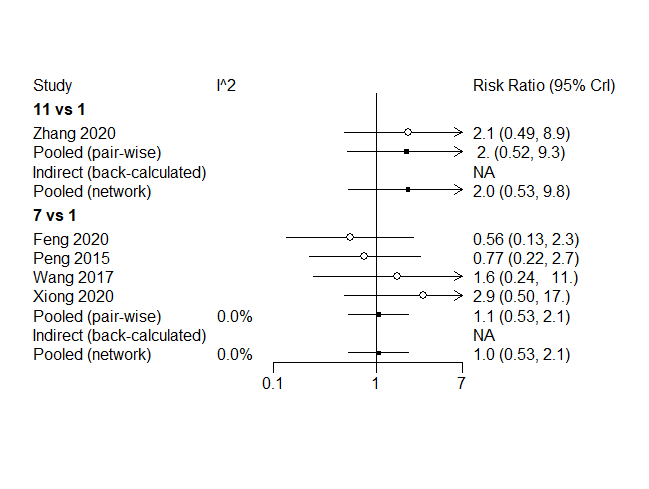


4.4.2 Adverse events (Analysis of heterogeneity)


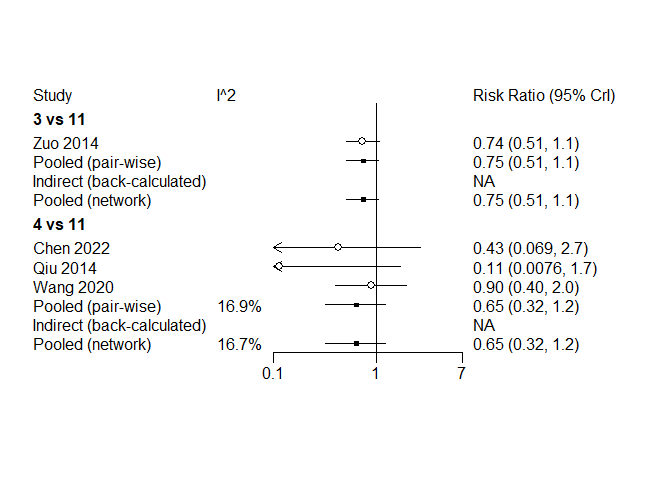


4.4.3 Adverse events (Analysis of heterogeneity)


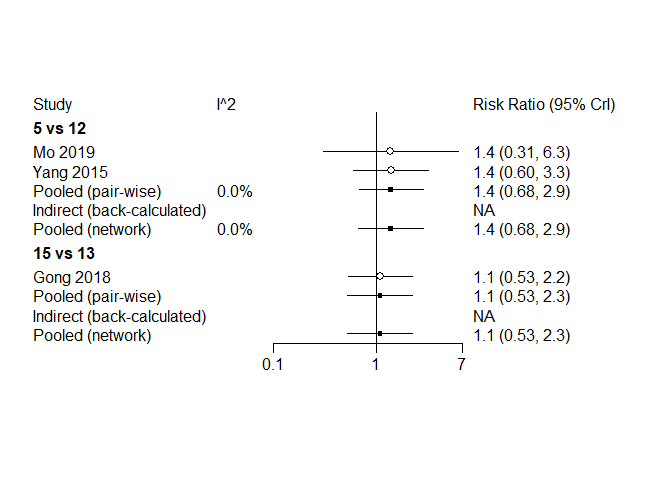


4.4.4 Adverse events (Analysis of heterogeneity)


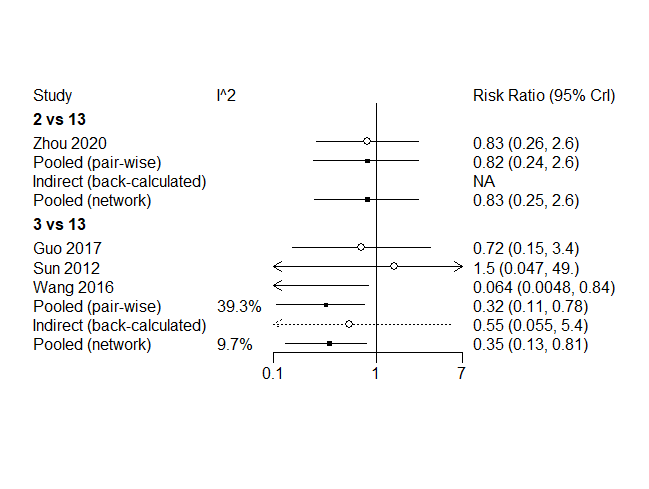


4.4.5 Adverse events (Analysis of heterogeneity)


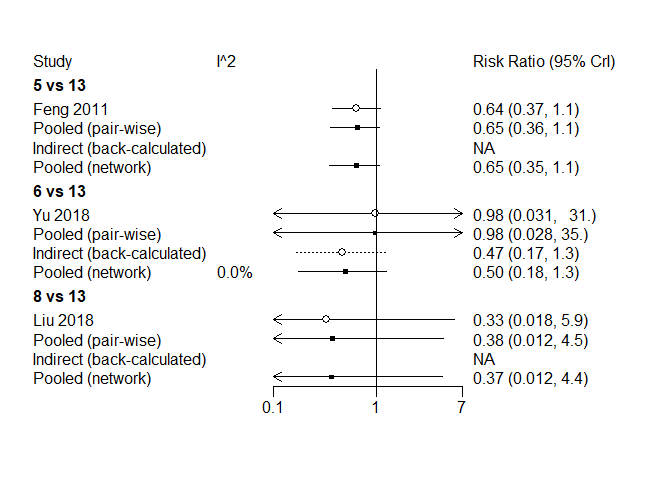


4.4.6 Adverse events (Analysis of heterogeneity)


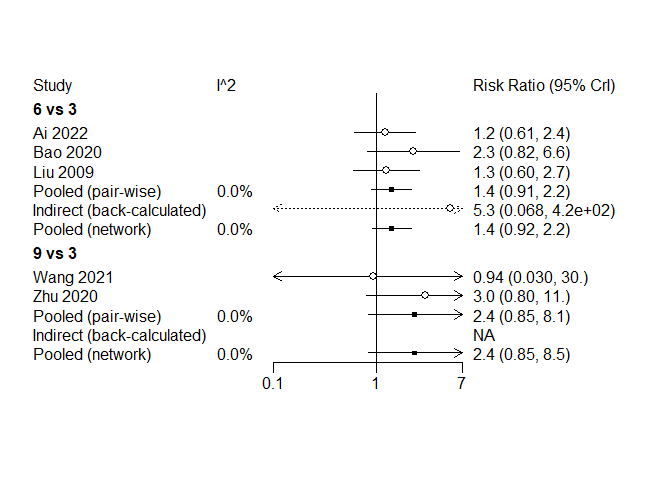


**Table S1:** Manufacturer、Specification and Medication of TWM

| **Study** | **Manufacturers** | **National Drug Approval Number** | **Ingredient** | **Method of taking medication**  **(po)** |
| --- | --- | --- | --- | --- |
| Wang 2016 | Hunan Qianjin Cooperation Pharmaceutical Co. | Z43020138 | TWM, 10mg/tablet | 40mg 3 times/d for 3 months, extend to 6 months if no remission, then taper to 20-30mg/d for 1 year |
| Feng 2020 | Zhejiang Duende Pharmaceutical Co. | Z33020422 | TWM, 10mg/tablet | 20mg/dose, 3 times/d, all before meals |
| Liang 2020 | Hunan Qianjin Cooperation Pharmaceutical Co. | Z43020138 | TWM, 10mg/tablet | 60mg/d, 3 times/d, gradually reduce the drug dose after 6 months of continuous treatment |
| Wang 2021 | Hunan Qianjin Cooperation Pharmaceutical Co. | Z43020138 | TWM, 10mg/tablet | 20mg/dose, 3 times/d |
| Ma 2014 | Jiangsu Meitong Pharmaceutical Co. | NR | NR | 20mg/dose, 3 times/d |
| Wang 2020 | NR | NR | NR | Initial dose of 40mg/dose, 3 times/d, reduced to 60mg/d after 3 months of treatment |
| Mo 2019 | Zhejiang Duende Pharmaceutical Co. | Z33020422 | TWM, 10mg/tablet | 20mg/dose, 3 times/d, tapered after 12 weeks |
| Zhu 2020 | Suizhou Yifan Pharmaceutical Co. | Z34021048 | TWM, 10mg/tablet | 1mg/kg·d, 3 times/d, after 2 months, start to reduce the dose by 10% every 2 weeks, and continue to take the dose of 10-20mg/d for 6 months |
| Guo 2017 | Yuanda Pharmaceutical Huangshi Feiyun Pharmaceutical Co. | Z42021212 | NR | 120mg·d^-1^ in 3 divided doses before meals for 3 months, reduced to 60mg·d^-1^ when patient achieves complete remission for up to 12 months |
| Zhang 2020 | Shanghai Fudan Fuhua Pharmaceutical Co. | Z31020415 | NR | Initial dose 30mg，3 times/d, after 12 weeks 20mg/d, 3times/d |
| Chen 2022 | Zhejiang Pulokongyu Natural Medicine Co. | Z33020778 | TWM, 10mg/tablet | 20mg/time, 3 times/d, all before meals |
| Sun 2012 | NR | NR | NR | 2mg-(kg-d)^-1^, divided into 3 oral doses, reduced maintenance after 3 months, discontinued if not effective for 6 months |
| Ai 2022 | Shanghai Fudan Fuhua Pharmaceutical Co. | Z31020415 | NR | Initial dose of 120 mg/d, reduced to 60 mg/d after 3 months, for 6 months |
| Zhou 2020 | Zhejiang Pulokongyu Natural Medicine Co. | Z33020778 | TWM, 10mg/tablet | Starting dose of 20 mg tid, tapered to a maintenance dose of 10 mg tid after 2 weeks |
| Bao 2020 | Zhejiang Duende Pharmaceutical Co. | Z33020422 | TWM, 10mg/tablet | 3 times/d, 40 mg/dose, 3 months of continuous treatment |
| Xue 2019 | Zhejiang Pulokongyu Natural Medicine Co. | Z33020778 | TWM, 10mg/tablet | Oral before meals, 3 times/day, 4 tablets/dose |
| Liu 2018 | Zhejiang Duende Pharmaceutical Co. | Z33020422 | TWM, 10mg/tablet | 1 mg-kg^-1^·d^-1^ orally 3 times daily |
| Gong 2018 | NR | NR | NR | 2mg/(kg·d) 3 times a day, maintain for 3 months and then reduce the dose to 1mg/(kg·d) |
| Zhang 2017 | Zhejiang Pulokongyu Natural Medicine Co. | Z33020778 | TWM, 10mg/tablet | 1 time/d for 3 months  (1 course of treatment), 120mg orally daily |
| Feng 2011 | NR | NR | NR | 20mg/time, 3 times/day, reduce the dose by half after 3 months of treatment |
| Liu 2009 | Taizhou Pharmaceutical Factory | NR | TWM, 10mg/tablet | 120mg/d orally in 3 divided doses before meals, and reduce the dose to maintenance dose  (60mg/d) for 3 months to achieve complete remission and continue until 12 months. If only partial remission is achieved in 3 months or if the treatment is ineffective, the dose can be extended to a maximum of 6 months and then reduced to 60mg/d for maintenance until 12 months. |
| Ding 2021 | Hunan Qianjin Cooperation Pharmaceutical Co. | Z43020138 | TWM, 10mg/tablet | 2 tablets/time, 3 times/d, gradually reduce the dosage after 6 months of continuous use |
| Xiong 2020 | Zhejiang Duende Pharmaceutical Co. | Z33020422 | TWM, 10mg/tablet | 60mg/d orally in 3 divided doses before meals for 6 months, then gradually reduce the dose until discontinuation |
| Wang 2017 | NR | NR | NR | 60 mg/d, 3 times/day, before meals, after 6 months of continuous use, gradually reduce the drug dose until discontinued |
| Yang 2015 | Lunan Houpu Pharmaceutical Co. | Z37020344 | NR | 1.0mg/kg orally 3 times daily |
| Peng 2015 | Zhejiang Duende Pharmaceutical Co. | Z33020422 | TWM, 10mg/tablet | 60mg-d^-1^ orally before meals in 3 divided doses, gradually reduced to discontinued after 6 months |
| Qiu 2014 | NR | NR | NR | 60mg/d |
| Xie 2021 | Hunan Qianjin Cooperation Pharmaceutical Co. | Z43020138 | TWM, 10mg/tablet | 20mg/dose, 3 times/d |
| Yu 2018 | Hunan Qianjin Cooperation Pharmaceutical Co. | Z43020138 | TWM, 10mg/tablet | 20mg/dose, 3 times/d |

**Table S2:** Adverse events reported in the included studies

| Study | Treatment-adverse events | Gastrointestinal symptoms  (mainly nausea,vomiting, diarrhea, and epigastric discomfort) | Liver function damage  (including elevated serum ALT/AST, etc.) | Bone marrow suppression  (mainly leukopenia, anemia, and thrombocytopenia) | Infections  (including respiratory tract infection, urinary tract infection, skin infection, etc.) | Glucose tolerance  (abnormal glucose metabolism, new-onset diabetes) | Others  (serum creatinine doubling, thrombosis, new hypertension, dizziness, hypotension, dry mouth, etc.) | Menstrual disorder, amenorrhea, decreased sperm count |
| --- | --- | --- | --- | --- | --- | --- | --- | --- |
| Wang 2016 | GC+TWM | 0 | 0 | 0 | 0 | 0 | 0 | 0 |
| Wang 2016 | GC+CAA | 0 | 0 | 0 | 0 | 0 | 0 | 0 |
| Feng 2020 | CNI+TWM | 2 | 0 | 0 | 0 | 0 | 0 | 3 |
| Feng 2020 | CNI | 3 | 0 | 0 | 0 | 0 | 0 | 0 |
| Liang 2020 | CNI+TWM | NR | NR | NR | NR | NR | NR | NR |
| Liang 2020 | CNI | NR | NR | NR | NR | NR | NR | NR |
| Wang 2021 | GC+TWM | 0 | 0 | 0 | 0 | 0 | 1 | 0 |
| Wang 2021 | GC | 0 | 0 | 0 | 0 | 0 | 0 | 0 |
| Ma 2014 | GC+TWM | 0 | 0 | 0 | 0 | 0 | 0 | 0 |
| Ma 2014 | GC | 0 | 0 | 0 | 0 | 0 | 0 | 0 |
| Wang 2020 | GC+CNI+TWM | 5 | 2 | 0 | 0 | 2 | 0 | 0 |
| Wang 2020 | GC+CNI | 5 | 2 | 0 | 0 | 3 | 0 | 0 |
| Zuo 2014 | GC+TWM | 0 | 5 | 0 | 7 | 7 | 6 | 7 |
| Zuo 2014 | GC+CNI | 0 | 5 | 0 | 5 | 11 | 17 | 0 |
| Mo 2019 | GC+MMF/LEF+TWM | 1 | 1 | 1 | 0 | 1 | 0 | 0 |
| Mo 2019 | GC+MMF | 1 | 0 | 1 | 0 | 1 | 0 | 0 |
| Zhu 2020 | GC+TWM | 2 | 0 | 0 | 1 | 0 | 0 | 0 |
| Zhu 2020 | GC | 5 | 0 | 0 | 3 | 0 | 0 | 0 |
| Guo 2017 | GC+TWM | 3 | 0 | 0 | 0 | 0 | 0 | 0 |
| Guo 2017 | GC+CAA | 3 | 0 | 0 | 0 | 1 | 0 | 0 |
| Zhang 2020 | CNI+TWM | 3 | 0 | 0 | 0 | 0 | 0 | 0 |
| Zhang 2020 | GC+CNI | 4 | 0 | 0 | 2 | 0 | 0 | 0 |
| Chen 2022 | GC+CNI+TWM | 1 | 1 | 0 | 0 | 0 | 0 | 0 |
| Chen 2022 | GC+CNI | 2 | 1 | 1 | 0 | 0 | 0 | 0 |
| Sun 2012 | GC+TWM | 0 | 0 | 0 | 0 | 0 | 0 | 0 |
| Sun 2012 | GC+CAA | 0 | 0 | 0 | 0 | 0 | 0 | 0 |
| Ai 2022 | GC+TWM | 7 | 2 | 2 | 0 | 3 | 0 | 0 |
| Ai 2022 | TWM | 10 | 4 | 1 | 0 | 2 | 0 | 0 |
| Zhou 2020 | GC+CAA+CNI+TWM | 1 | 2 | 2 | 0 | 0 | 0 | 0 |
| Zhou 2020 | GC+CAA | 2 | 1 | 3 | 0 | 0 | 0 | 0 |
| Bao 2020 | GC+TWM | 3 | 1 | 0 | 0 | 1 | 0 | 0 |
| Bao 2020 | TWM | 5 | 4 | 0 | 0 | 2 | 0 | 0 |
| Xue 2019 | GC+TWM | NR | NR | NR | NR | NR | NR | NR |
| Xue 2019 | TWM | NR | NR | NR | NR | NR | NR | NR |
| Liu 2018 | GC+CNI+MMF+TWM | 0 | 0 | 0 | 0 | 0 | 1 | 0 |
| Liu 2018 | GC+CAA | 2 | 0 | 0 | 0 | 0 | 0 | 0 |
| Gong 2018 | CAA+MMF+TWM | 6 | 2 | 4 | 0 | 0 | 0 | 0 |
| Gong 2018 | GC+CAA | 8 | 1 | 2 | 0 | 0 | 0 | 0 |
| Zhang 2017 | GC+MMF/LEF+TWM | NR | NR | NR | NR | NR | NR | NR |
| Zhang 2017 | TWM | NR | NR | NR | NR | NR | NR | NR |
| Feng 2011 | GC+MMF/LEF+TWM | 4 | 3 | 0 | 0 | 0 | 0 | 2 |
| Feng 2011 | GC+CAA | 4 | 0 | 5 | 2 | 0 | 0 | 2 |
| Liu 2009 | GC+TWM | 3 | 3 | 0 | 0 | 1 | 3 | 0 |
| Liu 2009 | TWM | 5 | 4 | 0 | 0 | 0 | 3 | 0 |
| Ding 2021 | GC+CNI+TWM | NR | NR | NR | NR | NR | NR | NR |
| Ding 2021 | GC+CNI | NR | NR | NR | NR | NR | NR | NR |
| Xiong 2020 | CNI+TWM | 1 | 1 | 0 | 0 | 0 | 0 | 0 |
| Xiong 2020 | CNI | 2 | 3 | 0 | 0 | 0 | 0 | 0 |
| Wang 2017 | CNI+TWM | 1 | 1 | 0 | 0 | 0 | 0 | 0 |
| Wang 2017 | CNI | 1 | 2 | 0 | 0 | 0 | 0 | 0 |
| Yang 2015 | GC+MMF/LEF+TWM | 2 | 3 | 4 | 0 | 0 | 2 | 0 |
| Yang 2015 | GC+MMF | 2 | 1 | 3 | 0 | 0 | 2 | 0 |
| Peng 2015 | CNI+TWM | 2 | 1 | 0 | 0 | 0 | 0 | 2 |
| Peng 2015 | CNI | 1 | 0 | 0 | 1 | 2 | 0 | 0 |
| Qiu 2014 | GC+CNI+TWM | 0 | 0 | 0 | 0 | 0 | 0 | 0 |
| Qiu 2014 | GC+CNI | 0 | 0 | 0 | 2 | 0 | 3 | 0 |
| Xie 2021 | GC+TWM | NR | 0 | 0 | 0 | 0 | NR | 0 |
| Xie 2021 | GC | NR | 0 | 0 | 0 | 0 | NR | 0 |
| Yu 2018 | TWM | 0 | 0 | 0 | 0 | 0 | 0 | 0 |
| Yu 2018 | GC+CAA | 0 | 1 | 0 | 0 | 0 | 0 | 0 |

**Table S3:** The anti-PLA2R antibody outcome reported in included studies

|  | Group 1 | | | | Group 2 | | | |  |
| --- | --- | --- | --- | --- | --- | --- | --- | --- | --- |
| Study ID | Treatment | Sample Size | Mean | Standard Deviation | Treatment | Sample Size | Mean | Standard Deviation | Units |
| Feng 2020 | CNI+TWM | 26 | -2.36 | 0.52 | CNI | 26 | -0.84 | 0.78 | μg/L |
| Wang 2021 | GC+TWM | 25 | -335 | 106.52 | GC | 25 | -261 | 103.32 | μmol/L |
| Guo 2017 | GC+TWM | 42 | -2.36 | 0.51 | GC+CAA | 42 | -0.84 | 0.79 | ρ/μg·L^-1^ |
| Ding 2021 | GC+CNI+TWM | 45 | -2.8 | 0.17 | GC+CNI | 45 | -0.9 | 0.3 | RU/mL |
| Xie 2021 | GC+TWM | 13 | -40.39 | 29.79 | GC | 13 | -25.58 | 25.84 | RU/mL |
| Yu 2018 | TWM | 34 | -336.81 | 118.98 | GC+CAA | 33 | -222.64 | 135.89 | μmol/L |
